# Quillworts from the Amazon: A multidisciplinary populational study on *Isoetes serracarajensis* and *Isoetes cangae*

**DOI:** 10.1371/journal.pone.0201417

**Published:** 2018-08-08

**Authors:** Gisele Lopes Nunes, Renato Renison Moreira Oliveira, José Tasso Felix Guimarães, Ana Maria Giulietti, Cecílio Caldeira, Santelmo Vasconcelos, Eder Pires, Mariana Dias, Maurício Watanabe, Jovani Pereira, Rodolfo Jaffé, Cinthia Helena M. M. Bandeira, Nelson Carvalho-Filho, Edilson Freitas da Silva, Tarcísio Magevski Rodrigues, Fernando Marino Gomes dos Santos, Taís Fernandes, Alexandre Castilho, Pedro Walfir M. Souza-Filho, Vera Imperatriz-Fonseca, José Oswaldo Siqueira, Ronnie Alves, Guilherme Oliveira

**Affiliations:** 1 Environmental Genomics Group, Instituto Tecnológico Vale, Belém, PA, Brazil; 2 Environmental Geology and Water Resources Group, Instituto Tecnológico Vale, Belém, PA, Brazil; 3 Biodiversity and Ecosystems Services Group, Instituto Tecnológico Vale, Belém, PA, Brazil; 4 Environmental Technology Group, Instituto Tecnológico Vale, Belém, PA, Brazil; 5 Botany Coordination, Museu Paraense Emílio Goeldi, Belém, PA, Brazil; 6 Zoobotanical Park, Vale, Parauapebas, PA, Brazil; 7 Environmental Studies, Amplo Engenharia, MG, Brazil; 8 Environmental Studies Office, Vale, Belo Horizonte, MG, Brazil; 9 North Ferrous Environmental Office, Vale, Parauapebas, PA, Brazil; 10 Director, Instituto Tecnológico Vale, Belém, PA, Brazil; Universita degli Studi di Siena, ITALY

## Abstract

*Isoetes* are ancient quillworts members of the only genus of the order Isoetales. The genus is slow evolving but is resilient, and widespread worldwide. Two recently described species occur in the Eastern Brazilian Amazon, *Isoetes serracarajensis* and *Isoetes cangae*. They are found in the ironstone grasslands known as Canga. While *I*. *serracarajensis* is present mostly in seasonal water bodies, *I*. *cangae* is known to occur in a single permanent lake at the South mountain range. In this work, we undertake an extensive morphological, physiological and genetic characterization of both species to establish species boundaries and better understand the morphological and genetic features of these two species. Our results indicate that the morphological differentiation of the species is subtle and requires a quantitative assessment of morphological elements of the megaspore for diagnosis. We did not detect differences in microspore output, but morphological peculiarities may establish a reproductive barrier. Additionally, genetic analysis using DNA barcodes and whole chloroplast genomes indicate that although the plants are genetically very similar both approaches provide diagnostic characters. There was no indication of population structuring *I*. *serracarajensis*. These results set the basis for a deeper understanding of the evolution of the *Isoetes* genus.

## Introduction

*Isoetes* is one of the most enigmatic plant groups and is the single living representative genus of the Isoetales order. The Isoetales order is well represented in the fossil records and its forms range from the gigantic arborescent individuals from the Carboniferous period (e.g. *Lepidodendrum* and *Sigillaria*) to smaller unbranched forms in the Paleozoic (e.g. *Chaloneria*) and Triassic periods (e.g. *Pleuromeia* and *Annalepis*) [1]. The genus is currently globally distributed and comprises approximately 250 species [2]. *Isoetes* is morphologically quite simple and the plants consist of a lobed subterranean bulb (corm) producing a downward tuft similar to a shoot axis, from which lateral organs named rootles develop (known as stigmarian roots), and upwards leaves with most species having four air-chambers. The genus is heterosporous, differing on the origin of the split from the mother spore and contact with each other, with monolete microspores and trilete megaspores [2]. Habitat, habit, colour, size, and ornamentation of the mega- and microspore, the proportion of the sporangium wall covered by the velum and the sporangial wall colouration are some of the most useful characters in the taxonomy of the genus [3]. However, habitat adaptations during the evolutionary process of these plants appear to have led to the morphological simplicity, convergence, parallel or reticulate evolution, and reversion, which result in great difficulties for species identification [4, 5].

**Fig 1:**
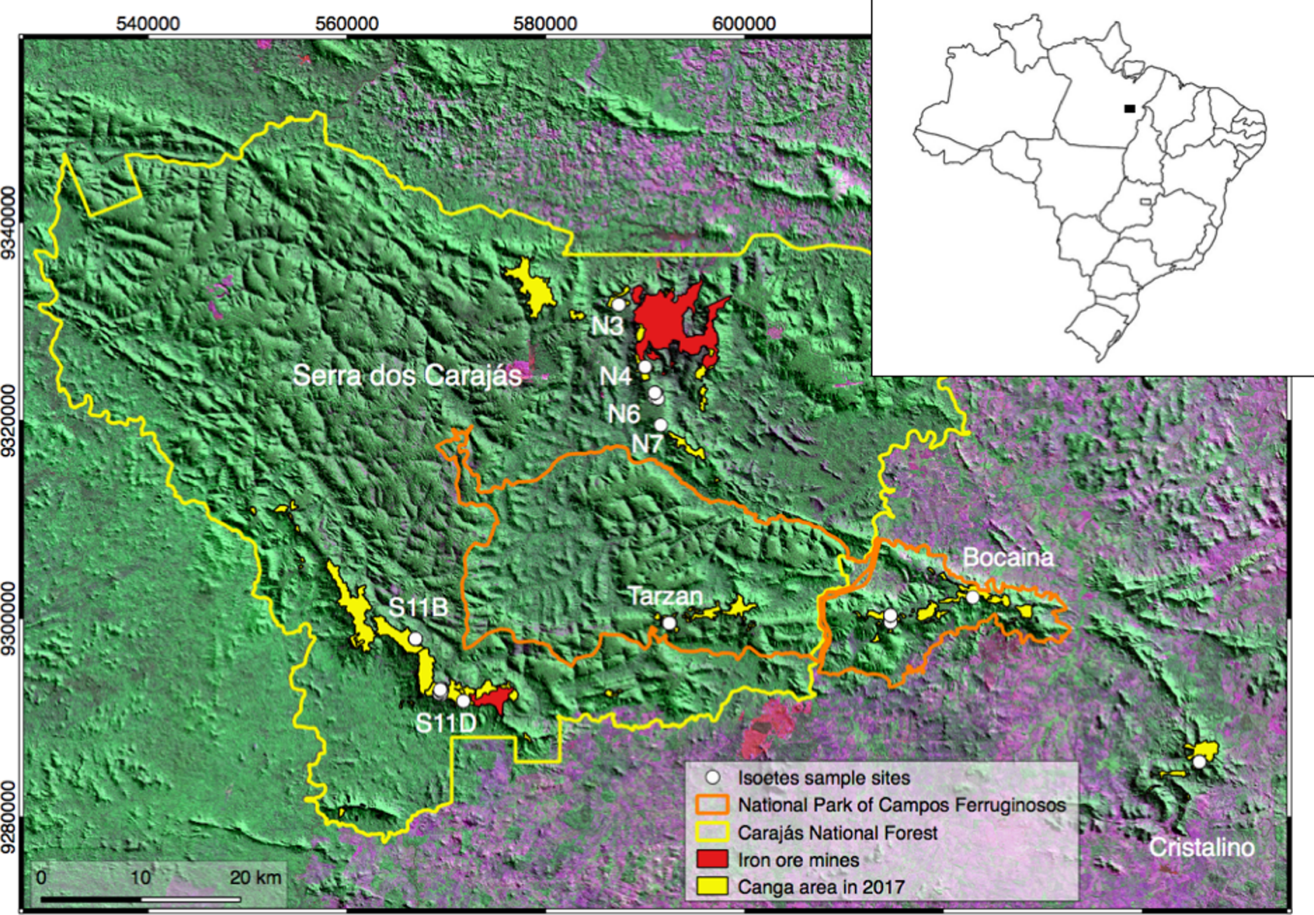
Map of occurrence of plants of the *Isoetes* genus in the region of Carajás. The map indicates the area of the Carajás National Forest and of the recently created National Park of Campos Ferruginosos. The location of mines is coloured in red and the canga areas in yellow. The locations where *Isoetes* were collected are indicated in the Serra Norte (N3-N7) and Serra Sul (S11B-S11D) (see also S1 Table). The black square on the map of Brazil points to the study site.

**Fig 2:**
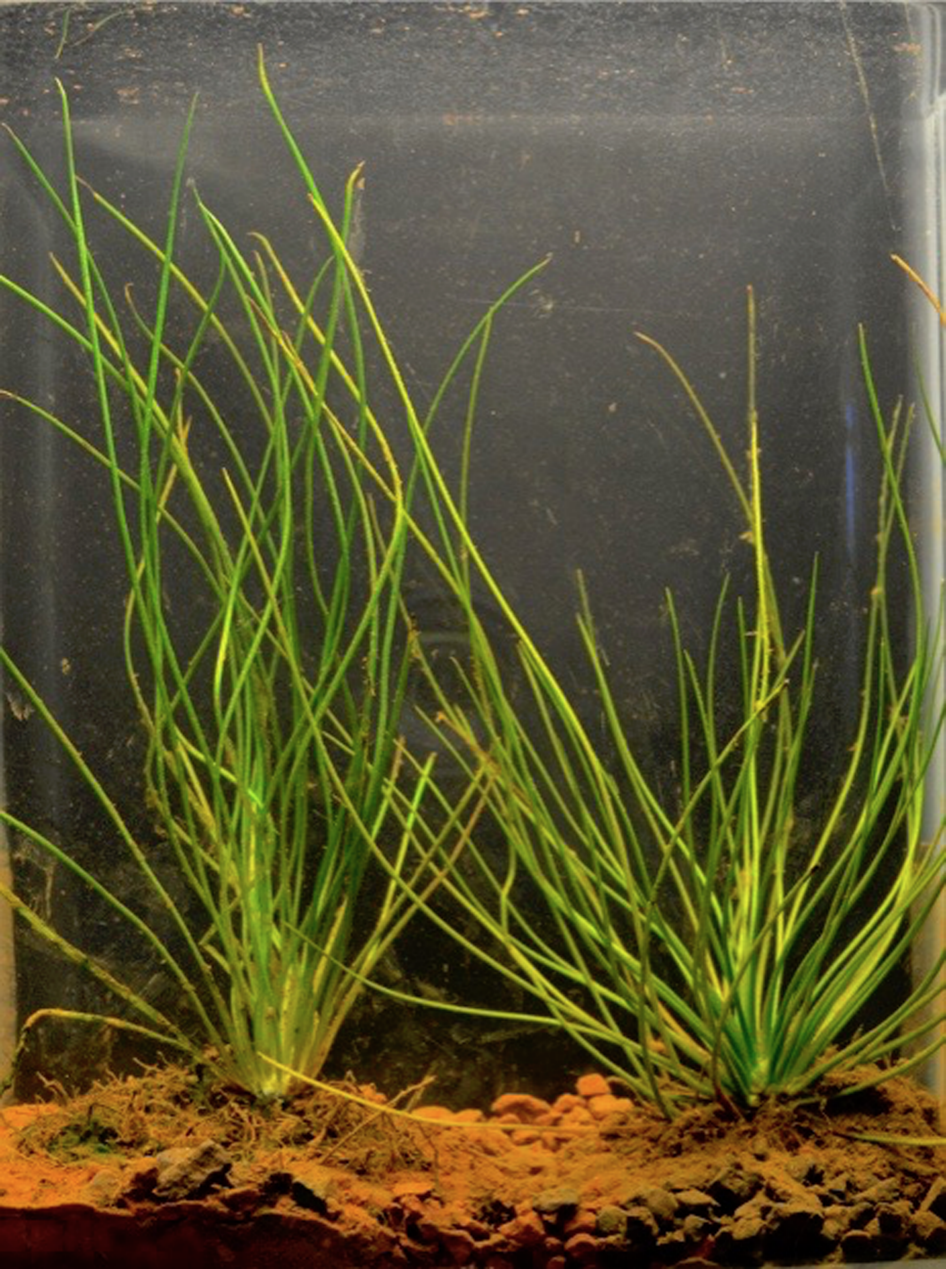
Photo of *Isoetes serracarajensis* (left) and *Isoetes cangae* (right), both maintained in the laboratory.

Two new *Isoetes* species were recently described from the Cangas of the Serra dos Carajás, Pará, in the Brazilian Amazon [6, 7]. The Serra dos Carajás is comprised of north and south ranges (Serra Norte and Serra Sul, respectively), located above 700 m in altitude [8]. Two national parks were created by the Federal Government of Brazil that include part of the Carajás mountains, the Carajás National Forest and the National Park of Campos Ferruginosos (Ferruginous Fields) (Fig 1), that include significant areas of Canga that hosts a number of endemic plant species just recently being studied in fine detail [9]. Both Carajás *Isoetes* are aquatic, but *I*. *serracarajensis* can survive in seasonal lakes and ponds, while *I*. *cangae* occurs submerged in a single natural lake (Fig 2) [7].

The *Isoetes* species are morphologically very similar to each other and usually, a small number of specimens are studied for the characterization of a new species. The species description is essentially based on megaspore morphology, with additional features described on the sporangium [7]. Species differentiation in the genus *Isoetes* is based on minute morphological attributes, likely the result of a very slow molecular evolution rate [10–13]. This underscores the fragility of relying on morphological characteristics for wide biodiversity assessments.

For those groups that display overlapping morphological characters among populations, DNA based approaches are important in assisting taxonomic decision as the discriminating criteria [14]. Therefore, methods such as DNA barcoding or high-resolution chloroplast or nuclear genome sequencing approaches have a huge potential to reveal more efficient molecular markers for species characterization and identification [15–17]. In this study, we aim to describe in detail morphological, physiological and genetic characteristics of the two species. The results will contribute to conservation efforts and to the understanding of the evolution of this ancient taxonomic group.

## Material and methods

### Biological material collection

*Isoetes* specimens were sampled in the Serra dos Carajás, in the State of Pará in the Brazilian Amazon. Fig 1 indicates the localities where 165 specimens were collected based on prior research conducted in herbariums worldwide and on local observations (Tables 1 and S1). *I*. *serracarajensis* plants were collected and kept in the Plant Growth laboratory at ITV (Instituto Tecnológico Vale). In general, the samples were collected at the margins of grassland hydromorphic areas and shallow water lakes.

### Morphometric analysis of the leaves

A total of 28 specimens of *Isoetes* representing a set of three wild populations were scored for the morphometric and morphological analysis. From the Serra Sul at the Carajás National Forest 11 specimens of *I*. *cangae* were obtained. Specimens of *I*. *serracarajensis* were collected from two temporary water bodies (eight and nine specimens from S11B and S11D, respectively, Fig 1). The specimens were collected at least 5 m from each other (Tables 1 and S1).

**Table 1:** Summary of specimens collected for genetic and morphological analyses. Please see S1 Table for a complete list of specimens and locations.

Species	City	Sector	Number of specimens
*Isoetes serracarajensis*	Parauapebas	N3	10
		N4WS	14
		N6	20
		N7	6
	Canaã dos Carjás	Bocaina	26
		Tarzan	10
		S11B	17
		Cristalino	10
		S11D	6
*Isoetes cangae*	Canaã dos Carajás	S11D	47

Tarzan—Serra do Tarzan, Bocaina—Serra da Bocaina. Region indicates the location of sampling (see Fig 1).

A data matrix was constructed with three linear measurements of the leaves. The selected characters were length, width at the base, and width at the middle of the leaves. We calculated the arithmetic mean to obtain a final value based on three measurements per specimen. The characters were measured with a ruler or digital pachymeter.

We conducted multivariate analyses using R and SYSTAT 10.0 to assess morphological variability and the reliability of the characters based on length and width of the sporophylls for species identification (SYSTAT Software). Ordination methods and cluster analysis were used to spot the affinities among populations and assess whether morphological patterns emerged from analyzes. Cluster analysis was performed using the unweighted pair group algorithm with arithmetic average (UPGMA) and standardized data based on Euclidean distance matrix obtained for all populations.

### Palynological analyses

Seven specimens of *I*. *cangae* were collected in the Amendoim Lake at S11D. Nine specimens of *I*. *serracarajensis* were collected in temporary ponds covered by water only during the rainy season, from S11D and S11B at Serra Sul (Fig 1 and Tables 1 and S1). The megaspore data were based on measurements of 20 spores extracted from each sporangium. One set was untreated and a second set chemically treated with 40% HF for removing the siliceous outer coating [18]. Subsequently, the megaspores were transferred to aluminum scanning electron microscope (SEM) stubs coated with a gold alloy and digitally imaged using a Zeiss SIGMA VP. EDS (Energy Dispersive Spectroscopy) was also carried out for megaspores with and without silica coating. The morphometric data were based on measurements of 20 spores and were plotted in box plots using Statistica 12 (Statsoft., 2015). The relation between equatorial diameter length (El) and equatorial width (Ew) was indicated by El/Ew, while the relation between polar (P) and equatorial (E) diameter was indicated by P/E. TLI (Trilete index: radius length / (trilete spore diameter/2)) was measured to indicate the relation between the radius and the spore diameter. All terminologies followed Punt et al. (2007) and Hickey (1986) [19, 20].

Microspores sizes and number per sporange were obtained from intact microsporangia gently removed from outer sporophylls of *I*. *cangae* (six sporangia from three plants) and *I*. *serracarajensis* (four sporangia from three plants) grown under laboratory conditions. A block of soil substrate from the temporary ponds of Serra Sul at Carajás was brought to the Plant Growth Lab at ITV. The plants were submerged in a container filled with distilled water and kept inside a growth chamber (Fitotron® SGC 120, Weiss Technik UK, Loughborough, United Kingdom) under 100 μmol m-2s-1 radiation, photoperiod of 12h:12h and day/night temperature of 26/22°C. Microsporangia were collected six months later after plant emergence when sporophylls reached around 10–12 cm. Spores were released from the sporangia with scalpels and rinsed with distilled water to remove debris. The number of microspores per sporangia was determined cytometrically by adding it into BD TruCount Absolute Count Tubes (BD Biosciences) on a FACSAria II (BD Biosciences). Microspores sizes (polar and equatorial diameter) were measured using a Zeiss Axio Imager M2 light microscope equipped with a Zeiss Axio Cam MRm camera.

### DNA extraction

Plant DNA extraction was carried out using a QIAcube HT robot (Qiagen), with approximately 50 mg of plant material collected in NaCl-saturated CTAB solution [21]. Samples were weighed and transferred to 2 mL Safe-Lock Eppendorf microtubes containing two 5 mm stainless steel beads (Qiagen). Subsequently, the tubes were frozen in liquid nitrogen and the leaf material was pulverized using a TissueLyser II (Qiagen) for 2 min at 30 Hz. After this 1 mL of extraction buffer [2% CTAB, 0.1 mM Tris-HCl (pH 8.0), 20 mM EDTA (pH 8.0), 1.4 M NaCl] was added to the samples, which were kept in a water bath at 60°C for 40 min under gentle agitation. The tubes were then centrifuged for approximately 30 s at 14,000 rpm and 200 μL of the supernatant was transferred to a 96 sample S-block. Afterwards, DNA extraction was carried out using the QIAamp 96 DNA kit (Qiagen), following the manufacturer’s instructions. DNA quantity and quality were checked using the Eon spectrophotometer (Bioteck).

### DNA barcoding

DNA barcoding was performed targeting the nuclear ITS2 and the cpDNA intergenic spacers *trn*H-*psb*A. The markers *atp*F-*atp*H and psbK-psbI were also used for a few specimens (S1 Table). All PCR reactions were conducted as follows: 2 μL of genomic DNA, 2.5 μL of 10X reaction buffer [100 mM Tris-HCl (pH 8.3) and 500 mM KCl], 2.4 μL of 25 mM MgCl_2_], 2 μL of dNTP mix (2 mM each), 0.5 μL of each forward and reverse primers at 10 pmol (Table 2), 1 U of Taq polymerase (Thermo Fisher) and milli-Q water to a final volume of 25 μL [22]. The PCRs were run in a Veriti 96-Well Thermal Cycler (Thermo Fisher), using the following conditions: 94°C for 3 min, followed by 30 cycles of amplification with 1 min at 94°C, 1 min at 54°C and 1 min at 72°C, with a final extension step for 7 min at 72°C. The amplified DNA was then precipitated with 100 μL of 65% isopropanol for 15 min, and centrifuged for 45 min at 4,000 rpm at 10°C. After discarding the supernatant, 125 μL of cold 70% ethanol was added and the tubes were centrifuged for 10 min at 4,000 rpm at 10°C. Subsequently, the supernatant was discarded, dried at room temperature (ca. 20°C) for 30 min and the DNA was resuspended in 25 μL of milli-Q water and stored at -20°C.

**Table 2:** List of primers used for PCR amplification of genetic markers.

Marker	Primer Name	Sequence 5’-3’	Reference
ITS2	ITS-S2F	ATGCGATACTTGGTGTGAAT	[23]
	ITS3R	GACGCTTCTCCAGACTACAAT	
trnH-psbA	trnH	CGCGCATGGTGGATTCACAATCC	[24]
	psbA	GTTATGCATGAACGTAATGCTC	
atpF-atpH	atpF	ACTCGCACACACTCCCTTTCC	[24]
	atpH	GCTTTTATGGAAGCTTTAACAAT	
psbK-psbI	psbK	TTAGCCTTTGTTTGGCAAG	[24]
	psbI	AGAGTTTGAGAGTAAGCAT	

Sequencing reactions were carried out using the BigDye Terminator v3.1 kit (Thermo Fisher) following the manufacturer’s protocol and then submitted to sequencing using an ABI 3730 Genetic Analyzer (Thermo Fisher).

Editing and assembly of the sequences were carried out using Geneious R10 (Biomatters). Electropherograms produced by Sanger sequencing were quality-trimmed using the modified Mott algorithm considering the chance of error of 0.01 per base. The contigs were assembled based on the overlapping of the quality trimmed bases (Phred >20) and the fasta files were generated for further analysis and deposit in the BOLD (BOLD http://www.boldsystems.org) and GenBank databases (https://www.ncbi.nlm.nih.gov). GenBank accession numbers for DNA barcodes are: atpF-atpH (MF805103—MF805125), ITS2 (MF805393—MF805555), trnH-psbA (MF805006—MF805102) and psbK-psbI (MF804937—MF804950). BOLD accession numbers are: ISO001-17 –ISO047-17, ISO049-17 –ISO064-17, ISO067-17, ISO070-17 –ISO072-17, ISO074-17 –ISO079-17, ISO081-17, ISO084-17 –ISO174-17.

Taxonomic affiliation of the sequences was based on the traditional taxonomy of each specimen compared with data deposited in public databases retrieved by BLASTn (http://blast.ncbi.nlm.nih.gov/Blast.cgi) [25]. Sequence alignments were conducted using MAFFT v7.309 with default parameters [26] using Geneious R10 [27].

### Whole genome sequencing

Total genomic DNA was extracted from leaves according to the CTAB I protocol as previously described [28]. Shotgun sequencing runs were performed using two different methodologies. Sequencing on the Ion Torrent Personal Genome Machine® (PGM™) Sequencer (Thermo Fisher) was performed by using total genomic DNA (1 μg) sheared with the Bioruptor Plus sonication device (Diagenode) and the sequencing library prepared according to the Ion Plus Fragment Library kit following Ion Torrent PGM™ protocol (Thermo Fisher). The resulting individual DNA library was quality checked and quantified using the Qubit 2.0 Fluorometer and the Qubit dsDNA HS Assay Kit following the manufacturer’s specification (Thermo Fisher). Following template amplification and enrichment on the Ion OneTouch™ 2 System and Ion OneTouch™ ES enrichment system, respectively (Life Technologies). Next, samples were individually loaded onto one PGM Ion 318™ Chip v2 and sequenced using the Ion PGM™ Hi-Q™ Sequencing Kit (Thermo Fisher) according to manufacturer’s protocol. The NextSeq 500 Illumina platform was also used for sequencing ten *Isoetes* specimens. Briefly, paired-end libraries were constructed from ~50 ng of DNA. Samples were subjected to a step of enzymatic random fragmentation in which the DNA was simultaneously fragmented and bound to adapters using the QXT SureSelect kit (Agilent Technologies) according to the manufacturers' instructions. The fragmented DNA was purified and subjected to an amplification reaction using primers complementary to the adapters. Next, the libraries were quantified using the Qubit® 3.0 Fluorimeter (Life Technologies) and checked for fragments size in the 2100 Bioanalyzer (Agilent Technologies®). The libraries were diluted in a solution of 0.1% Tris-HCl and Tween and pooled. The sequencing run was performed within a NextSeq 500 v2 kit high-output (300 cycles). Sequencing output is summarized in S2 Table.

The reads with base quality < Phred 20 and length < 100 bp were trimmed and the remaining reads with more than 20% low-quality bases (<Phred 20) were filtered out using Fastx-Toolkit (http://hannonlab.cshl.edu/fastx_toolkit/). High-quality reads from each sample were then mapped against the reference chloroplast genome (*Isoetes flaccida* chloroplast, accession GU191333, [29]) using Geneious R10.

Mapped reads from each sample were submitted to an assembly using SPAdes with default settings [30]. We also used ARC (http://ibest.github.io/ARC/) to obtain chloroplast contigs by mapping the raw reads against the reference chloroplast genome using bowtie and then automatically assembling the mapped reads with SPAdes [31]. The SPAdes contigs and the ARC contigs were merged manually with Geneious R10, producing the complete chloroplast genome from each sample. Easyfig was used for synteny analysis [32]. Gene annotations were conducted using DOGMA and CPGAVAS [33, 34]. The quality of genome assemblies was evaluated by Quast v 4.3 using statistical metrics such as Genome fraction, GC (%), Nx, mismatch, and indels per 100 kb [35]. Chloroplast genome sequences were deposited in GenBank under accession numbers MG019393 for ITV3828 (*I*. *cangae*), MG019394 for ITV2008 (*I*. *cangae*), and MG019395 for ITV411 (*I*. *serracarajensis*). Raw data were deposited at NCBI’s Sequence Read Archive (SRA) with the following IDs ITV2008 (SRR6941192), ITV411 (SRR6941193), and ITV3828 (SRR6941194).

Alignments of full chloroplast genomes were generated in mVISTA using a Shuffle-LAGAN mode and a sliding window of 100 bp to detect the whole genome variation [36].

Mapping of Illumina reads to assembled chloroplast genomes was conducted using Bowtie2 [31] to generate SAM files. SAMtools [37] was used to generate the BAM and VCF files needed to carry out SNP analysis, conducted with SNPrelate from Bioconductor (https://bioconductor.org/) [38]. Detailed information including command lines used can be found in S1 File.

## Results

The collection sites indicate that *Isoetes* are well represented in the area, occurring from the north range (Serra Norte): N3, N4, N6 and N7 and in the south range (Serra Sul) S11B and S11D in addition to the Tarzan, Cristalino and Bocaina (part of the new National Park of Campos Ferruginosos) mountains (Fig 1).

### Leaf morphology reveals overlapping phenotypes

**Fig 3:**
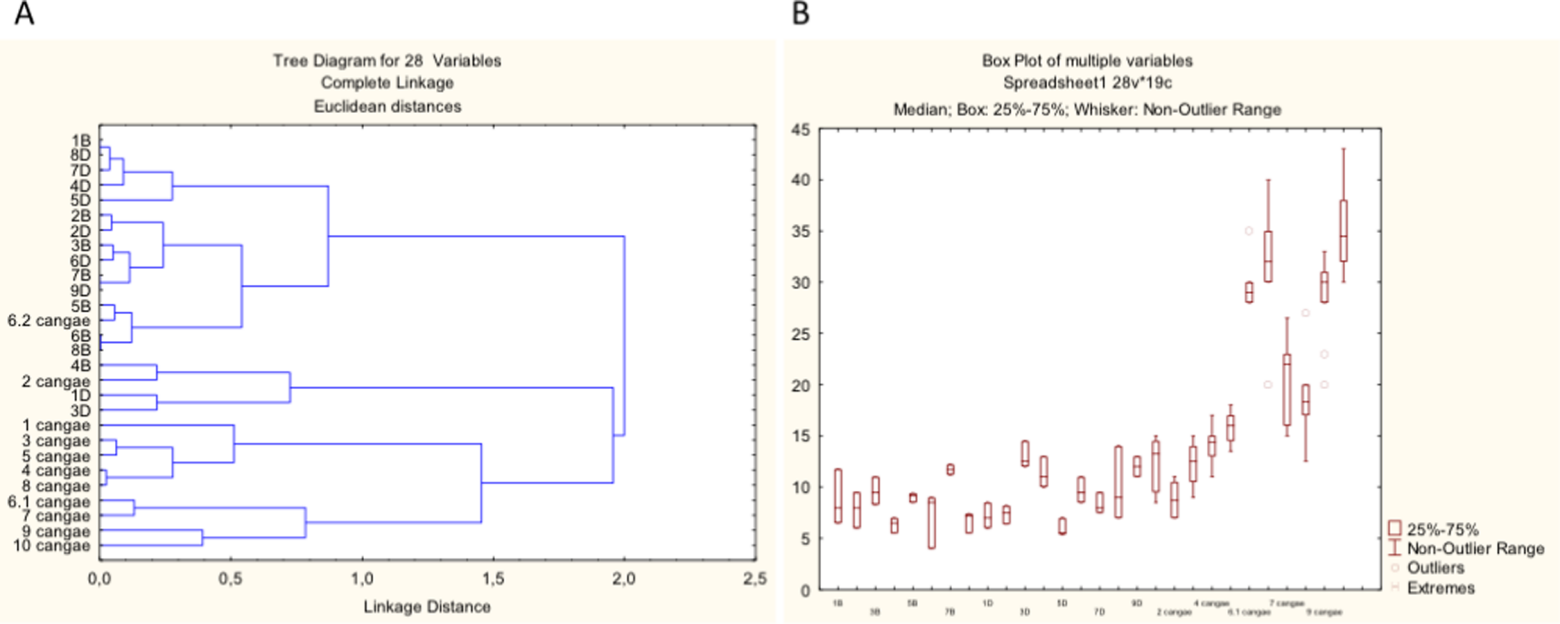
Leaf morphometric analysis. A) UPGMA dendrogram of the 28 specimens of the three populations of *Isoetes*. Data are based on Euclidean distances. Group of specimens: 1-8B, *I*. *serracarajensis* from S11B; 1-9D, *I*. *serracarajensis* from S11D; 1–10 cangae, *I*. *cangae* from Amendoin lake. B) Box-plot of multiple variables (Length, width in the base, width in the middle of the leaves) of three populations of *Isoetes*.

Cluster Analysis of leaf morphometry resulted in a dendrogram with two major clusters (Fig 3). The first (upper) cluster contains mostly specimens of *I*. *serracarajensis*, although one specimen of *I*. *cangae* appeared in this cluster. Specimens of *I*. *serracarajensis* from seasonal lakes at S11C and S11D form a mosaic with no distinctive patterns. Most specimens identified as *I*. *cangae* are represented in the lower cluster, in which two subgroups are formed: the major subgroup only contains specimens of *I*. *cangae*, and the upper subgroup containing three specimens of *I*. *serracarajensis* with one specimen of *I*. *cangae*. The box-plot in Fig 3 reveals a statistically skewed dataset of characters indicating a large spread of the data. Comparisons between datasets of *I*. *cangae* and *I*. *serracarajensis* indicate that the morphometric distribution of *I*. *serracarajensis* is more homogeneous than the distribution of *I*. *cangae*. Moreover, the superposition of leaf morphometry between the two species is evidenced (Fig 3). Therefore, larger leafs were observed for *I*. *cangae*, although measurements were not diagnostic for species identification. We next sought to analyze other morphological characters with a focus on megaspores that were initially used as the main feature to describe the two *Isoetes* species from Carajás.

### Megaspore ornamentation and PCA of morphometric characteristics differentiate the two *Isoetes* species

**Fig 4:**
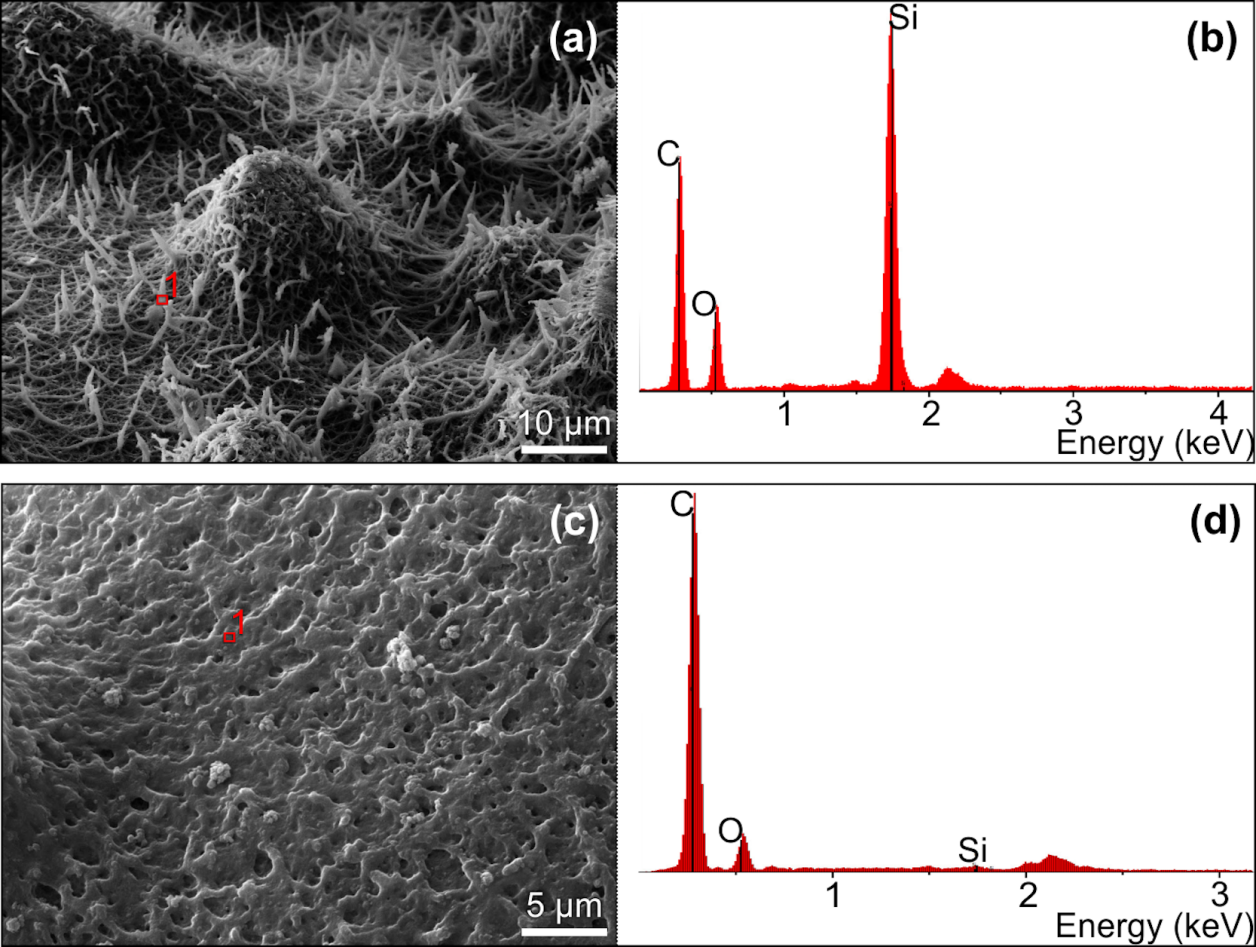
EDS Spectrum detecting silica in the megaspore surface of *Isoetes*, a) with silica and b) without silica coating.

One important characteristic of *Isoetes* megaspores is that they usually present a silica coat. This was demonstrated by EDS spectrum of the surface of *Isoetes* megaspores that revealed peaks related to Si and O, indicative of the presence of silica (Fig 4A and 4B). After silica removal, C and O peaks remain, while the silica peak disappeared (Fig 4C and 4D), permitting a clear analysis of micro and macro ornamentation patterns. We conducted the analysis of all morphometric parameters in samples “with (w/)” and “without (w/o)” silica coating.

**Fig 5:**
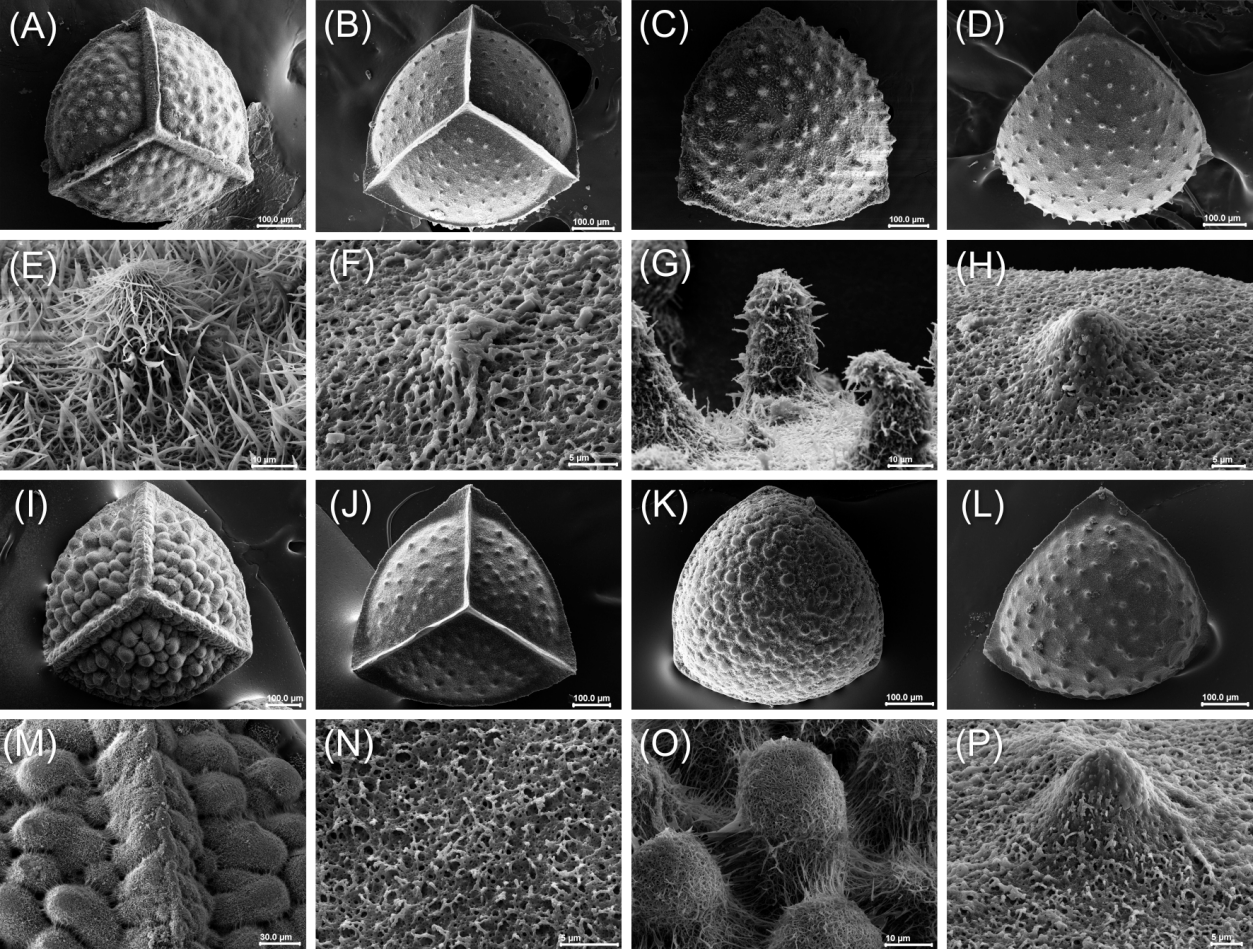
*Isoetes* megaspores (trilete mark on proximal face). A) Ic5: proximal face, verrucate megaspore (with silica); B) Ic7: proximal face, verrucate to tuberculate megaspore (without silica); C) Ic4: distal face, verrucate to tuberculate megaspore (with silica); D) Ic7: distal face, tuberculate to verrucate megaspore (without silica); E) Ic7, detail of the verruca and fused rodlets (with silica); F) Ic4: detail of the verruca and fused rootlet in the proximal face; G) Ic8: tubercles and fused rodlets in the proximal face (with silica); H) Ic7: detail of the verruca and fused rootlet (densely packed) in the proximal face (without silica); I) IsD8: proximal face, verrucate megaspore (with silica); J) IsD8: proximal face, verrucate megaspore (without silica); K) IsB2: distal face, verrucate megaspore (with silica); L) IsD2: distal face, verrucate megaspore (without silica); M) IsD1: detail of the radii and verrucae in the proximal face (with silica); N) IsB1, detail of the fused rootlets in the distal face (without silica); O) IsB5: detail of the verrucae and fused rodlets in the distal face (with silica); P) IsD2: detail of the verrucae and fused rootlet (densely packed) in the proximal face (without silica). Scale bar corresponds to 100 μm, except F, H, N and P, which is 5 μm. Ic: I. cangae, Is: *I*. *serracarajensis*, Is (B and D): *I*. *serracarajensis* from the S11B and S11D, respectively.

**Fig 6:**
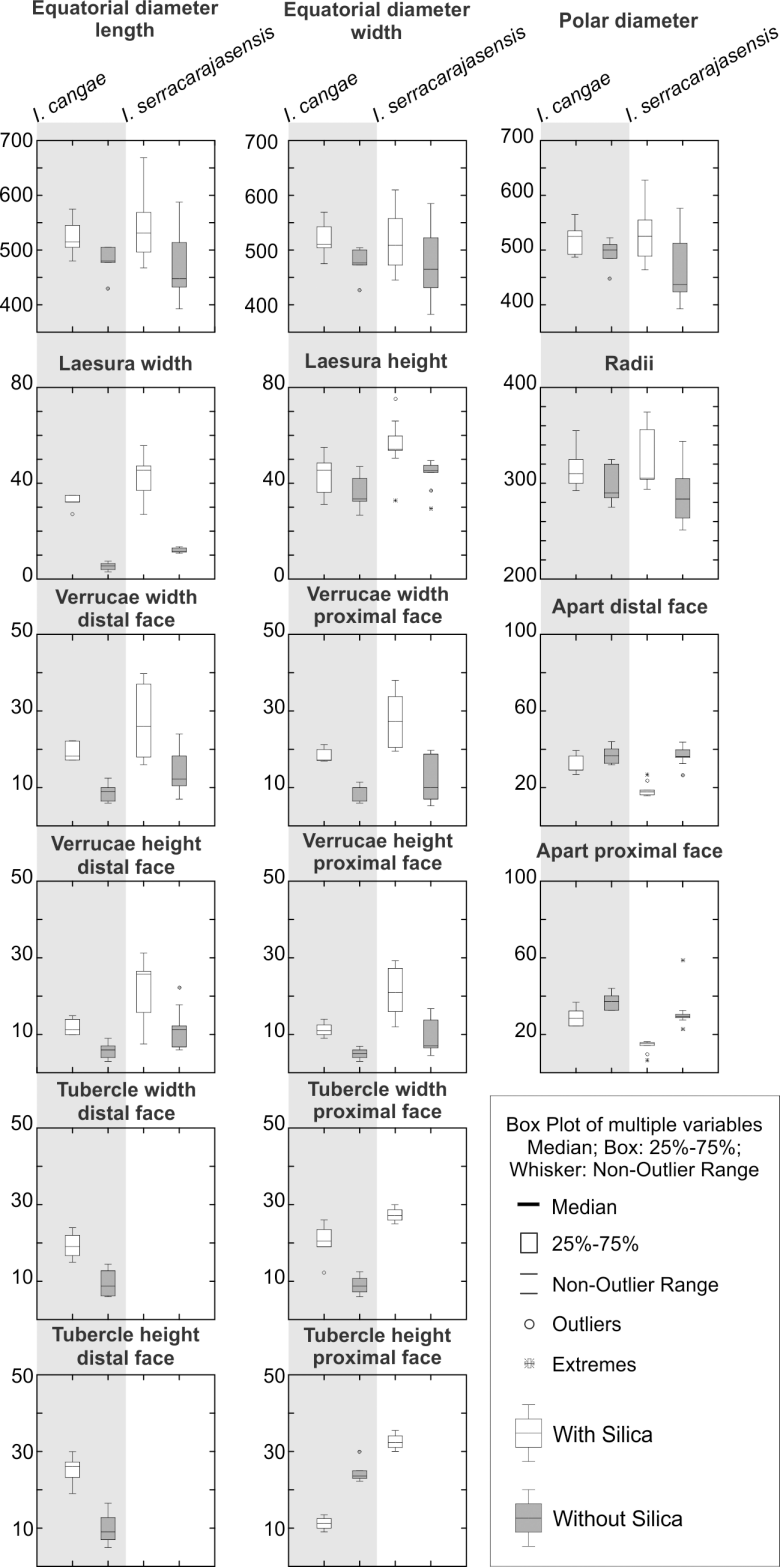
Box plots of *Isoetes* megaspore morphometry with (white boxes) and without silica (gray boxes). All units in the y-axis are in μm.

Megaspores of *Isoetes* are white if exposed, heteropolar and radially symmetric, with a triradiate (trilete) mark on the proximal face extending to the equatorial limb (Fig 5), which is evidenced by the TLI (radius length / (trilete spore diameter/2)) values > 1. Laesurae are prominent, generally wider than higher, and commissures are slightly curved to straight. Grooves and verrucae are present in some specimens of *I*. *serracarajensis*. Shapes are oblate spheroidal to prolate spheroidal (in samples with (w/) or without (w/o) silica) in equatorial view (polar/equatorial diameter—P/E 0.91–1.03) and triangular-obtuse to circular in polar view (equatorial length/width—El/Ew 0.99–1.07). Spores with P/E and equatorial length/width (El/Ew) < 1 or > 1 have acute to obtuse apex, except for *I*. *serracarajensis* that have an exclusively obtuse apex. Broadest ranges of El, Ew, and P were observed for *I*. *serracarajensis* (Table 3 and Figs 6 and S1). Wider and higher laesura occur for *I*. *serracarajasensis* (Table 3 and Figs 6 and S1). Considering megaspores w/, *I*. *cangae* is mainly verrucate to tuberculate (predominance of verrucae) with fused rodlets and rootlets in the distal and proximal face. *I*. *serracarajensis* is predominantly verrucate, rarely tuberculate, with fused rodlets and rootlets in the distal and proximal face. After silica removal, *I*. *serracarajensis* became entirely verrucate, and both species presented rootlets on both faces (Figs 5 and 6).

**Table 3:** Morphometric values (minimum–maximum) of *Isoetes* megaspores; w/ (with silica) and w/o (without silica).

Parameters	*I*. *cangae*		*I*. *serracarajasensis*	
	w/	w/o	w/	w/o
Equatorial diameter length—El	480–575	430–505	468–669	393–588
Equatorial diameter width—Ew	475–569	427–504	445–610	383–585
Polar diameter—P	487–565	447–522	464–628	393–576
Laesura width	27–35	3–7	27–56	11–14
Laesura height	31–55	27–47	33–75	29–50

**Fig 7:**
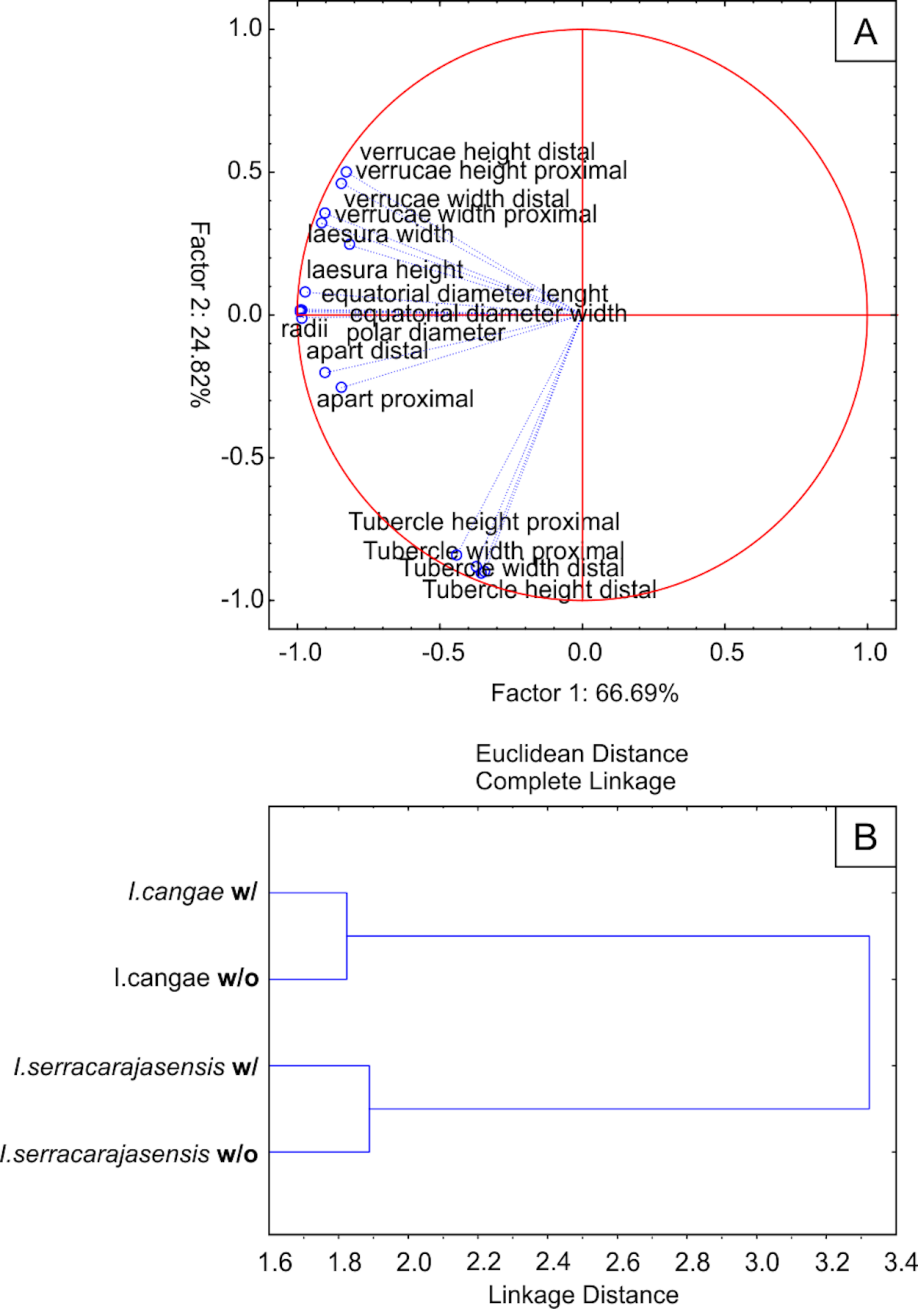
A) PCA of morphometric data of *Isoetes* megaspore showing tubercles group differentiated from other parameters (total variance: 91.5%). B) Distinct clustering of *I*. *cangae* from *I*. *serracarajasensis* with (w/) and without (w/o) silica coating.

The distance between verrucae is generally shorter for *I*. *serracarajensis*. The combination of all measured parameters resulted in distinct groups by PCA. A dominating factor for distinguishing *I*. *cangae* from *I*. *serracarajensis* was the presence of tubercles (negative loading on Factor 1, Fig 7). Silica removal influenced the result of PCA analysis, improving on the separation of the two species (Fig 7).

### Microspores show morphological differences but are produced in similar numbers

**Fig 8:**
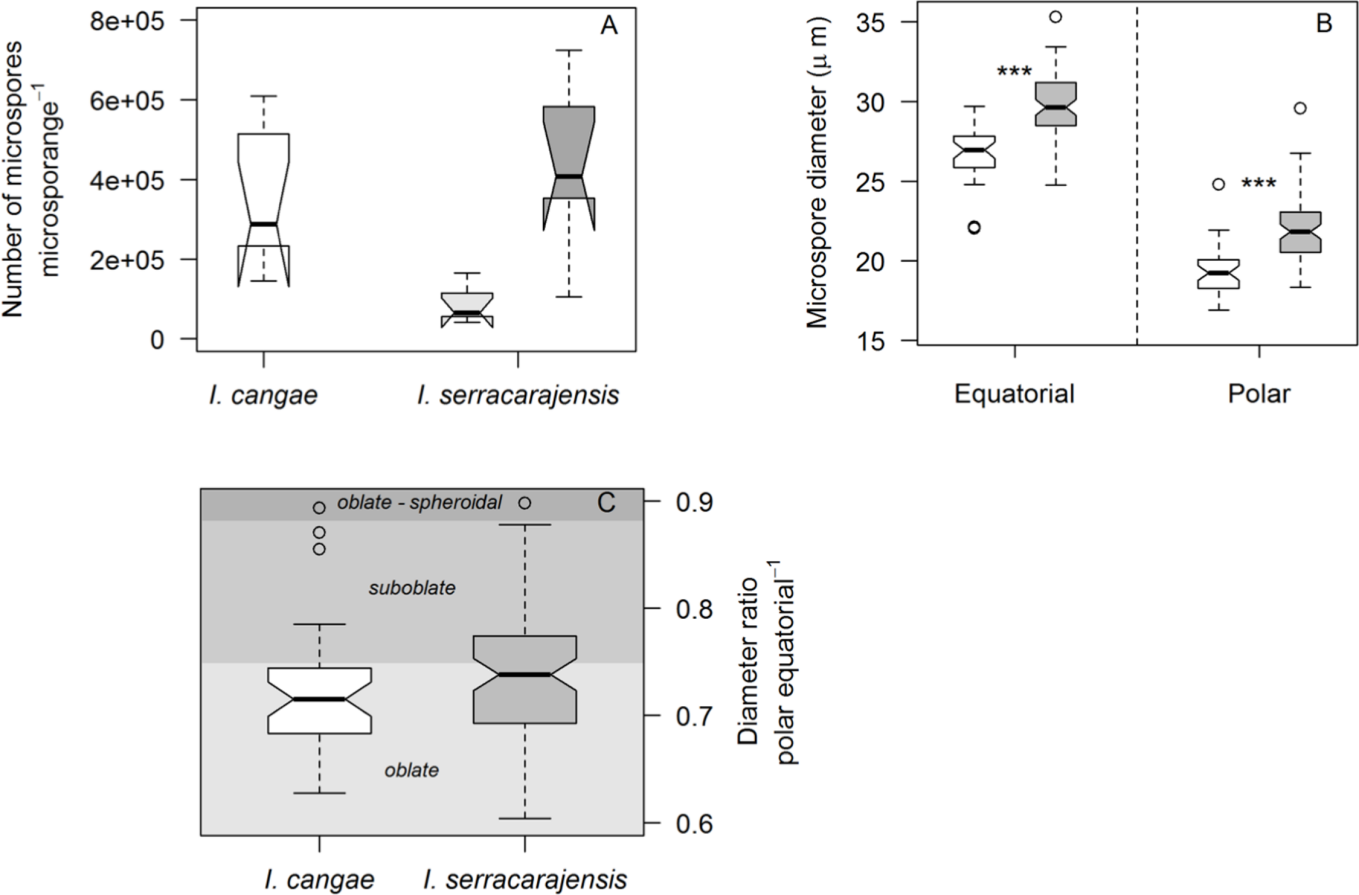
Microspore number and size of two *Isoetes* species from Carajás. A) Number of microspores per microsporangia. Light gray and dark gray represent two groups of *I*. *serracarajensis* independently collected, B) Microspore equatorial and polar diameters and, C) Polar/equatorial diameter ratio of *I*. *cangae* (six microsporangia from three plants) and *I*. *serracarajensis* (four microsporangia from three plants). The gray shades indicate different shapes. ‘***’ represents a significant difference in a t-test at P<0.01 and ‘ns’ non-significant.

We also sought to compare one physiological parameter between the two *Isoetes* species, the number of microspores produced. We observed that there was no difference in the number of microspores produced from the microsporangia at the base of each sporophyll leaf carrying a male sporangium between the two species (Fig 8A). We initially had collected *I*. *serracarajensis* specimens with significantly lower microspore production (Fig 8A, light gray), but more recently collected specimens displayed elevated microspore numbers making this parameter not a particular characteristic of the species (Fig 8A, dark gray). However, there were morphological differences. Microspores of *I*. *cangae* were smaller in equatorial and polar measurements (27 and 19 μm, respectively) in comparison to *I*. *serracarajensis* (30 μm and 22 μm, respectively), although this was not a diagnostic character (Fig 8B). We did not observe any significant difference in shape according to the descriptions of Erdtman [39] (Fig 8C).

### DNA barcoding identifies two distinct *Isoetes* populations

**Fig 9:**
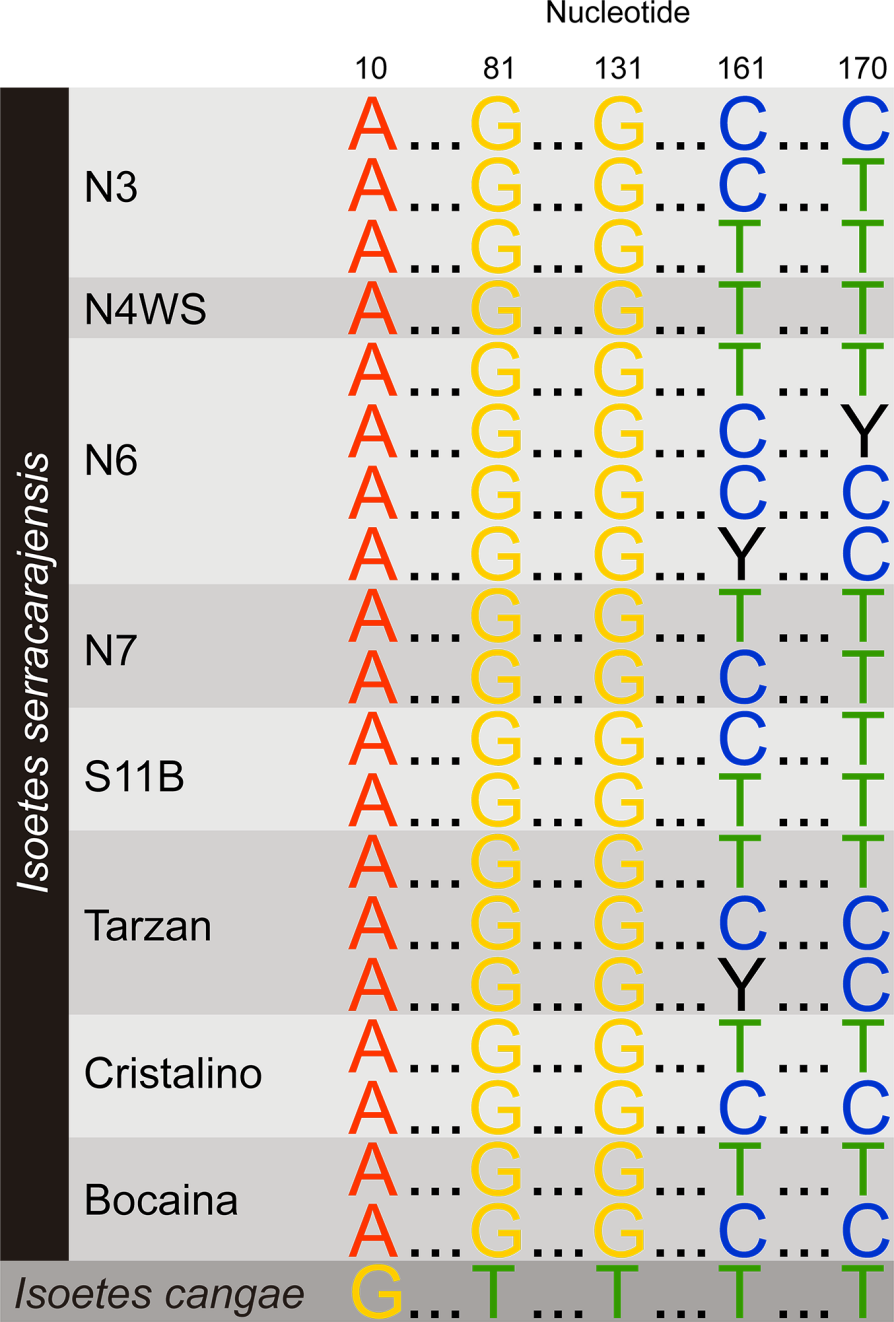
The ITS2 DNA barcode can be used to identify the two Carajás *Isoetes* species. Summary of the multiple alignment of all genotypes observed for both species. The site position is indicated on top. The site of origin of each genotype of *I*. *serracarajensis* is indicated. See S2 Fig for the complete multiple alignment.

We produced DNA barcodes for 165 specimens of *Isoetes* from both Carajás region species, distributed along a 100-kilometer range in different ironstone grasslands plateaus. The complete list of locations is available in S1 Table. All the tested chloroplast markers yielded identical sequences (data not shown). In contrast, the ITS2 nuclear ribosomal marker was capable of separating, at high confidence, *Isoetes* in two groups corresponding to the two Carajás species described (Fig 9). We observed that *I*. *cangae*, restricted to the S11D region, was separated from *I*. *serracarajensis* which was also observed in the same plateau area. Despite *I*. *serracarajensis* being widely spread in the region, we did not observe any differentiation among sampled plots, even considering the geographically distant Cristalino samples (Figs 9 and S2). To probe deeper at the genetic differences, we sequenced the plastid genomes of the two species.

### Chloroplast genomes are highly similar but contain distinguishing characteristics

**Fig 10:**
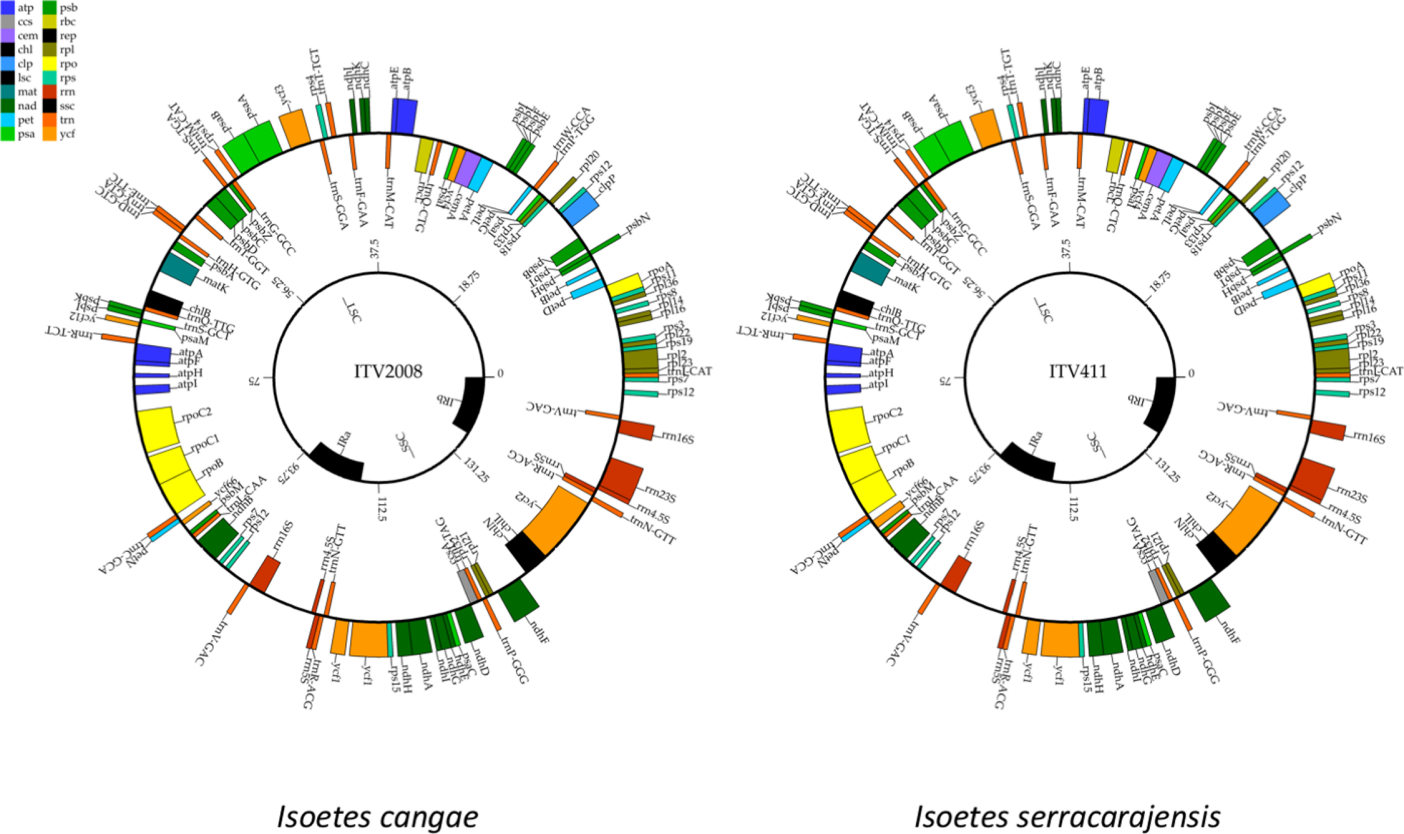
Complete chloroplast assembly for *I*. *cangae* and *I*. *serracarajensis*. Colors are indicative of the gene functions. The side of the circle indicates the transcribed DNA strand.

**Fig 11:**
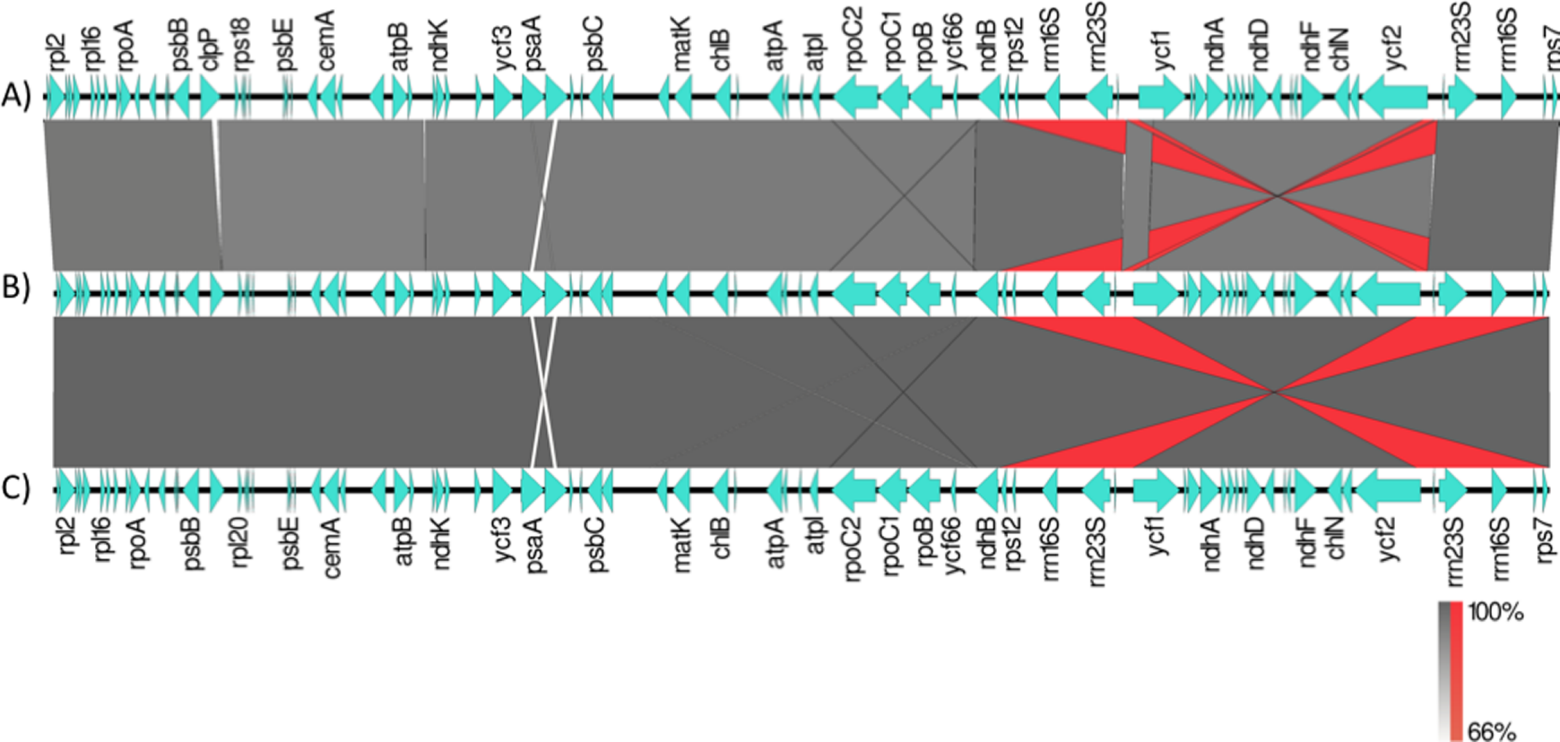
Synteny analysis *I*. *flaccida* (A), *I*. *serracarajensis* (B, ITV411) and *I*. *cangae* (C, ITV2008) chloroplast genomes. Genes are shown in light green with arrows pointing in the direction of transcription. Gray areas represent syntenic regions ranging from white to dark gray the greater the similarity between syntenic regions. Red represents repeated inverted regions.

The output generated by the whole genome sequencing is shown in S2 Table. The mapped reads of the *I*. *cangae* and *I*. *serracarajensis* against the reference genome of *I*. *flaccida* resulted in 79,820 and 75,008 mapped reads, respectively. The assembled plastids of *I*. *cangae* (189 X coverage) and *I*. *serracarajensis* (143 X coverage) were 143,415 bp (37.7% GC content) and 143,687 bp (37.7% GC content) long, respectively (Fig 10). The only other available chloroplast genome of the genus is of *I*. *flaccida* (NC_014675.1), which is 145,303 bp in length [29]. The gene content is identical between the two Carajás species, 130 genes, three less than what was observed for *I*. *flaccida* (S3 Table). The genes *rps*16, *rps*2 and *trn*K-UUU were missing from the Carajás species. Three copies of the *rps*12 gene and two copies of each *rps*7, *rrn*16S, *rrn*23S, *rrn*4.5S, *rrn*5S, *trn*A-UGC, *trn*L-GAU, *trn*N-GUU, *trnR-ACG* and *trn*V-GAC were observed. The genomes of the three species were completely syntenic with all genes in the same order and transcribed from the same DNA strand (Fig 10). One of the small differences between the Carajás species was a missing exon in the *clp*P gene, in addition to one intergenic fragment between the *atp*B and *ndh*K genes. The inverted repeats are shown in the synteny maps in red (Fig 11). We also observed small regions with few similarities between the species, such as the *psaA* and *psaB* genes (Fig 11).

**Fig 12:**
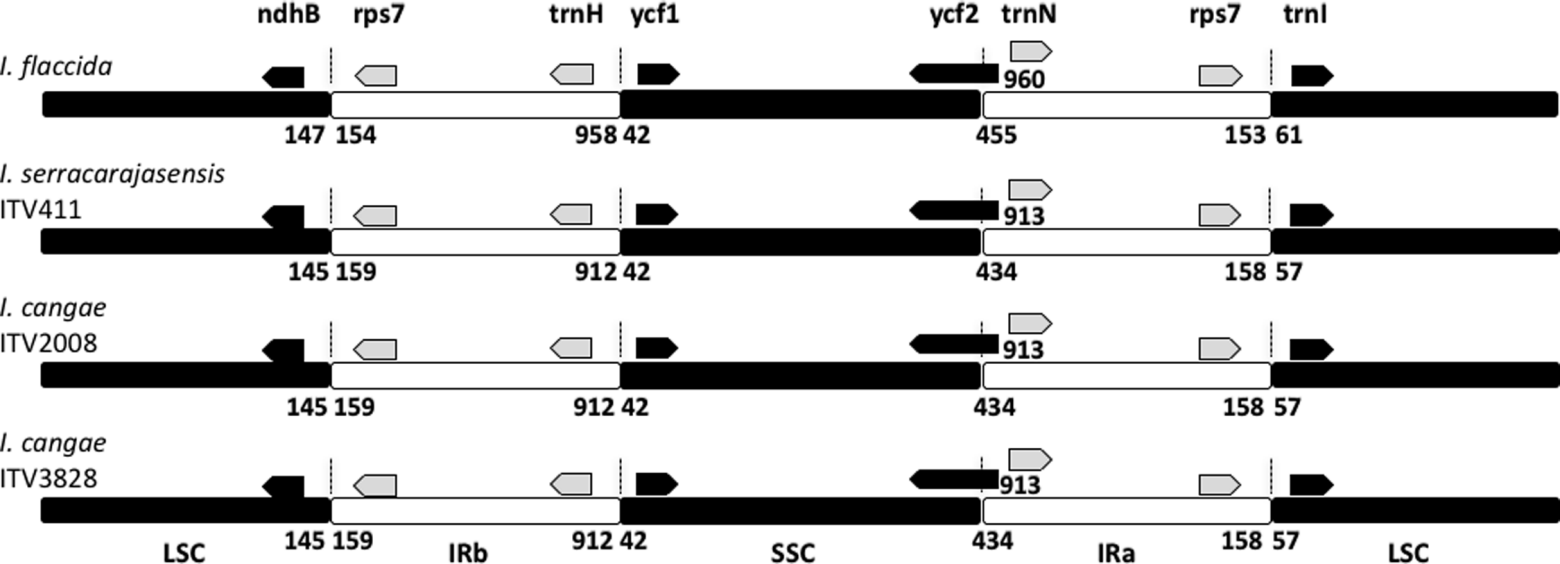
Inverted Repeat region of three different *Isoetes* species. Boxes with arrow indicate genes. The blocks in black or white represent the different repeats: LSC, IRb, SSC, and IRa.

Another representative aspect of the chloroplast structure were the boundaries between the Inverted Repeats. We observed that the number of bases between adjacent genes at the boundaries of the repeats LSC-IRb, Irb-SSC, SSC-IRa and IRc-LSC was equal between the Carajás *Isoetes* species and distinct from the boundaries observed for *I*. *flaccida* (Fig 12). Taken together, the overall chloroplast genomes of the three species were very similar, but with a greater similarity between the two species from Carajás.

**Fig 13:**
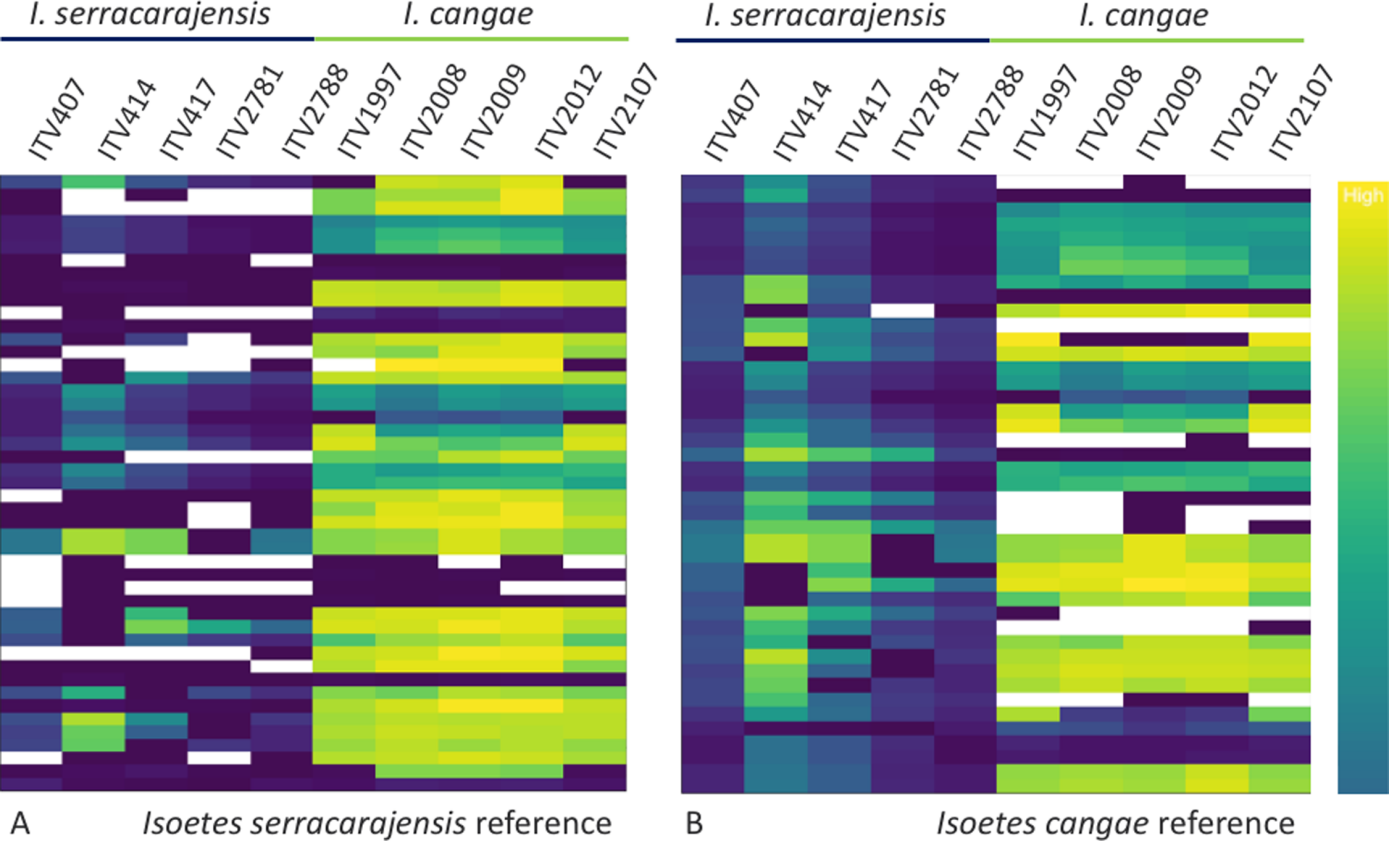
Sequence coverage of chloroplast SNPs separates the two *Isoetes* species. Illumina reads of the 10 sequenced chloroplasts were mapped to the reference genomes of *I*. *serracarajensis* (A) or *I*. *cangae* (B). Low coverage ranges from white to blue and high coverages range from green to yellow.

**Fig 14:**
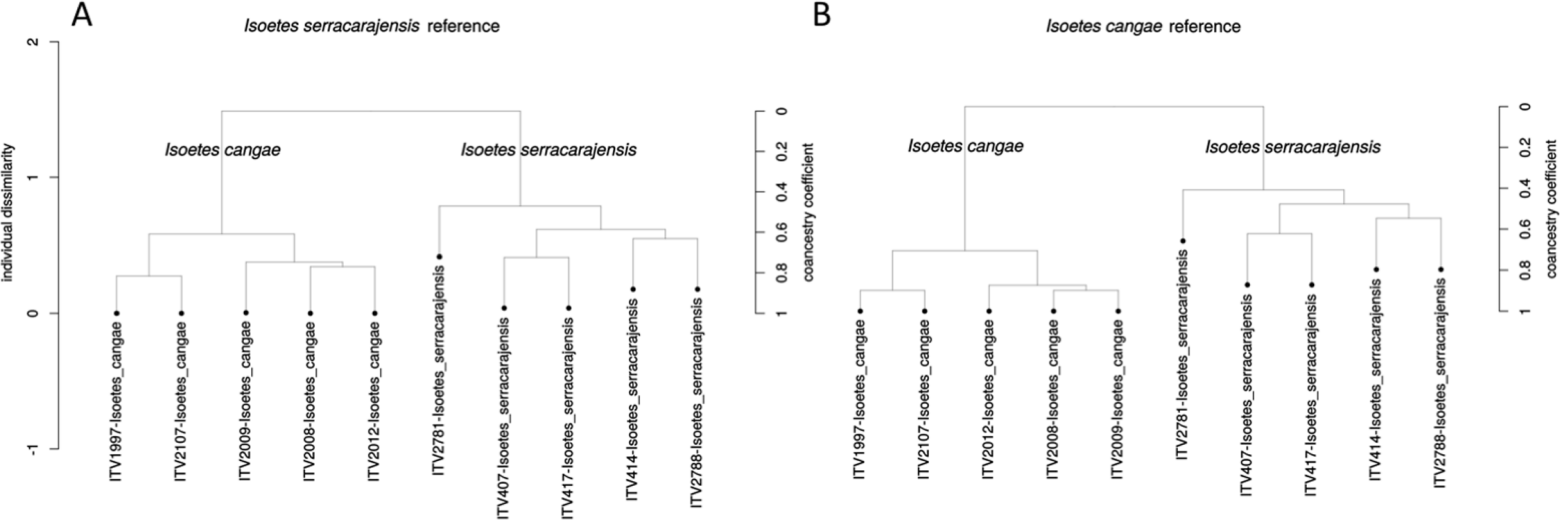
Individual dissimilarity trees based on SNP data separates the two *Isoetes* species. VCF SNP files were used by SNP Relate R package to measure coancestry coefficient.

To expand on the initial observation of the *I*. *cangae* and *I*. *serracarajensis* chloroplast genomes, 10 additional *Isoetes* genomes were sequenced from both species. The reads were mapped to the genomes of *I*. *cangae* or *I*. *serracarajensis*. When all genomes were mapped to the *I*. *serracarajensis* reference we observed 44 SNPs overall. There were 27 and 21 specific alleles for *I*. *serracarajensis* and *I*. *cangae*, respectively. From the 44 observed SNPs, only seven were in coding regions and all of them resulted in a switch in the coded amino acid (S4 Table). When the genomes were mapped to the *I*. *cangae* reference a total of 47 SNPs were observed. We observed 29 and 22 specific alleles for *I*. *serracarajensis* and *I*. *cangae*, respectively. From the 47 observed SNPs, only 13 were in coding regions and nine of them resulted in a switch in the coded amino acid (S5 Table). Codon usage in both chloroplasts did not show significant differences (S6 Table). Further analysis of the chloroplast genomes indicated that SNP analysis could differentiate the two species. For this analysis sequence coverage of the Illumina reads for each allele displayed by the reference genome is shown. Fig 13 indicates that coverage plots can group the plants of the same species. Trees based on SNP identification data with coancestry coefficient measures was also able to group plants of the same species by (Fig 14).

## Discussion

Our study reveals morphological and genetic information in an enigmatic and ancient group of plants. Chloroplast derived DNA barcodes could not separate both species and morphometric analyses was capable of species distinction only after extensive measurements and statistical analysis. However, ITS2 DNA barcodes and chloroplast genome analysis clearly placed the two *Isoetes* species in different clades, despite their close relatedness.

The characters used to describe the two Carajás *Isoetes* species were leaves and megaspores types. Despite the potential of leaf characters for taxonomy, leaf morphological aspects are not sufficient to distinguish those two species [40]. The leaf characters have been used with caution in the taxonomy of *Isoetes* and appear to be relevant to separate some species [41]. However, our analysis of the populations of *I*. *serracarajensis* and one population of *I*. *cangae* showed inconsistency in the distinction of these species based only on leaf measurements, with *I*. *cangae* displaying greater variability. This result was already expected given the huge morphological plasticity that the leave may present due to habitat variation [4]. This makes leaf morphometry useless for species identification, in this case.

Megaspores have also been used for the characterization of species [20]. We used a larger dataset for comparing silica coated megaspores of *I*. *cangae* and *I*. *serracarajensis* with those previously described [7]. Laesura was higher than wider and its shape was the same for both species, but grooves and verrucae were observed on each side of *I*. *serracarajensis*. Megaspore polar diameter and laesura width and height measurements were bigger than those previously described [7]. Taken together, the individual measurements made with silica-coated megaspores were not sufficiently clear to distinguish the two *Isoetes* species.

Considering that silica starts to accumulate from the tapetum after completion of the exospore [42, 43], the most appropriate way to describe macro and micro elements of ornamentation requires silica removal [18]. Troia and colleagues studied megaspores of Mediterranean *Isoetes* species and proposed that the ornamentation elements were still visible on the exospore even after silica removal, although in a reduced form [18]. Thus, the surface elements of the siliceous coating seem to conform to the sporopolleninous exospore as a deposit. Our results showed that silica coating partially conforms to the exospore since the order of predominance of the macro-ornamentation elements changed after silica removal, as well as the micro-ornamentation pattern. Thus, silica removal caused a significant reduction in all measurable features of the studied megaspores and did not improve qualitative distinction between the two species. However, PCA analysis did separate the two species and we believe that such statistical treatment will be necessary for the use of morphometric analyzes for diagnostic purposes. The approach is therefore of limited use in wide biodiversity assessments, but strongly necessary under stenopalynous condition.

One physiological parameter evaluated was the number of microspores produced. We also did not observe significant differences between the two species. The microspore shape, however, was distinct and is another evidence towards the segregation of the two *Isoetes* species. These species occupy contrasting environments, allowing them to develop different growth and reproductive strategies. Plants of *I*. *serracarajensis* can be found in temporary ponds, where a fine-tuned growth and reproduction in short periods of the rainy season (five months long) would have been beneficial. In contrast, plants of *I*. *cangae* inhabit a permanent lake that undergoes short variations of the environmental conditions all along the year [44]. Under these conditions, it is possible that growth and reproductive periods of *I*. *cangae* developed alternative reproductive patterns independent of the precipitation regularity. The *I*. *cangae* microspores were generally smaller, but this was not sufficiently distinct for a diagnostic character, but apparently provides a reproductive barrier for the species. The size of the microspores is related to the genome ploidy and as a consequence may block the exchange of genetic material between species, similar to what has been observed for polen [12, 45].

DNA sequence information based on the nuclear derived ITS2 DNA barcode could clearly place the two *Isoetes* species in different clades, despite their close relatedness. The sampled location for *I*. *serracarajensis* did not indicate any geographical stratification. Therefore, ITS2 DNA barcoding was sufficient for diagnosing the two species, but additional molecular markers are needed for capturing the phylogenetic relationships of *Isoetes* species [5].

Plastome genome variation in sequence, in inverted repeat length, and gene content has been observed in many plants [46, 47]. Ferns are an ancient lineage in which a number of differences have been described, including inversions and gene loss [48]. *I*. *cangae* and *I*. *serracarajensis* presented low levels of variation in the chloroplast genome, which also was captured by a lack of variability in DNA barcode markers. The complete chloroplast genomes corroborated the initial DNA barcode analysis showing a conserved genetic pattern between them. These species were similar in overall chloroplast genome architecture, gene content, gene order and spacing between repeat regions. On the other hand, the species of *Isoetes* from Carajás remarkably revealed three missing genes in comparison to *I*. *flaccida*: rps2, rps16 (both encode for ribosomal proteins of the small ribosomal subunit) and trnK-UUU (encoding for a ribosomal transfer gene). The *rps*16 gene has been observed to be absent in other chloroplast genomes, being substituted by the nuclear copy [49]. The *rps*2 and *rps*16 are pseudogenes in *I*. *flaccida* [30]. The *trn*K-UUU gene is one of the few transport RNA genes that contain introns. The *mat*K gene is usually found in this intron and is required for the *trn*K-UUU splicing [29, 50]. We manually checked to ascertain that *Trnk-*UUU was indeed missing in the Carajás species as well as in other *Isoetes*. The gene *trnk-*UUU has been observed to be missing in other species such as in leptosporangiate ferns [51].

The three missing genes in the Carajás *Isoetes* species appear to be consistent not only with the quite distinct phylogenetic origin but also with the ancient divergence of them from the clade of *I*. *flaccida* [5]. The divergence between Carajás *Isoetes* species and *I*. *flaccida* clades extends back to the earliest divergence event in *Isoetes* in the Late Jurassic ca. 148 Mya [5]. One interesting difference of *I*. *flaccida* from the Carajás species is that the inverted repeat boundaries display clear distinctions for this species in the boundaries length and could be another diagnostic trait. Further studies including the close relatives of Carajás *Isoetes* species will allow testing if these missing genes are widespread in their group and trace the evolutionary history of these species. In conclusion, this study is the first attempt to understand chloroplast genome differences between both closely and distantly related species in *Isoetes*. The overall structure of the chloroplast genomes is highly similar between species from the same clade, however, there are remarkable differences between species of distinct clades. It is clear that more species should be included in further analyses to better understand how the chloroplast architecture evolved in *Isoetes* and this study is an important step towards this goal.

To increase the resolution of the chloroplast genome analysis, we also searched for SNP variations among individuals of both species in the complete chloroplast genome sequence examination. Despite the occurrence of few polymorphisms, the SNP analysis could clearly place *I*. *serracarajensis* and *I*. *cangae* into two different groups. The phylogenetic analysis of the entire gene content showed the same results as the examination of DNA barcode and SNP data (data not shown).

We provide diagnostic molecular markers for the two species and information on the level of genetic variability in the region. The amount of genetic variability in a population and gene flow between populations is critical for the maintenance of a species. The molecular analysis corroborates the work of expert taxonomists, but also supports environmental assessment tasks by providing solid markers for species identification, especially for the non-expert [5, 7]. The information we provide contributes with baseline data to aid conservation efforts. We provide easy to produce markers for species identification and, for the moment, low-resolution markers for population genetics. *In situ* and *ex-situ* conservation efforts are facilitated by the low levels of genetic diversity observed for both *Isoetes* species.

We are currently conducting genome level analysis of several related species to capture their evolutionary history, full nuclear genomes, and population genetic analysis, of both Carajás species as well as paleobiogeographic reconstructions.

## Supporting information

S1 Table Specimens collected for genetic and morphological analyses. (DOCX)

S2 Table Statistics on NGS data for whole genome sequencing. (XLSX)

S3 Table Chloroplast gene content for *I*. *cangae* and *I*. *serracarajensis*. Genes were annotated in CPGAVAS [35], names in bold were annotated using DOGMA [34]. The copy number of each gene and transcribed strands are shown.
(XLSX)

S4 Table SNPs observed in the chloroplast genomes of specimens of *I*. *serracarajensis* and *I*. *cangae* mapped against ITV411 of *I*. *serracarajensis*. (XLSX)

S5 Table SNPs observed in the chloroplast genomes of specimens of *I*. *serracarajensis* and *I*. *cangae* mapped against ITV2008 of *I*. *cangae*. (XLSX)

S6 Table Codon usage for *Isoetes flaccida* and the Carajás species. (XLSX)

S1 File Bioinformatics command lines used. (DOCX)

S1 Fig Microspore measurements. Box plots of equatorial diameter length, equatorial diameter width, polar diameter, leasure width, leasura hight, and radii with TLI for *I*. *cangae* and *I*. *serracarajensis* with and without silica. All units in the y-axis are in μm.
(TIF)

S2 Fig Multiple alignment of the ITS2. The species and the sampled locations are shown in the left. Polymorphic sites are marked in green and yellow for *I*. *cangae* and in blue for *I*. *serracarajensis*.
(TIF)
