# Supplementary material for: Quillworts from the Amazon: A multidisciplinary populational study on Isoetes serracarajensis and Isoetes cangae
Source: PLoS One. 2018 Aug 8;13(8):e0201417. doi: 10.1371/journal.pone.0201417 (PMC6082551; doi:10.1371/journal.pone.0201417)
Supplement: S1 Table — (DOCX) [file pone.0201417.s001.docx]

Supplemental Table S1: Specimens collected for genetic and morphological analyses.

|  |  |  |  |  | **Molecular markers** | | | | | **Analysis** |
| --- | --- | --- | --- | --- | --- | --- | --- | --- | --- | --- |
| **BOLD ID** | **Sample ID** | **Species** | **City** | **Sector** | **ITS2** | **trnH-psbA** | **psbK-psbI** | **atpF-atpH** | **rpoC1** |  |
| ISO001-17 | ITV249 | IS | Parauapebas | N3 | x | x | x | x | - | Barcodes |
| ISO002-17 | ITV250 | IS | Parauapebas | N3 | x | x | x | x | - | Barcode |
| ISO003-17 | ITV251 | IS | Parauapebas | N3 | x | x | x | x | - | Barcode |
| ISO004-17 | ITV254 | IS | Parauapebas | N3 | x | x | - | x | - | Barcode |
| ISO005-17 | ITV255 | IS | Parauapebas | N3 | x | x | - | x | - | Barcode |
| ISO006-17 | ITV256 | IS | Parauapebas | N3 | x | x | - | x | - | Barcode |
| ISO007-17 | ITV257 | IS | Parauapebas | N3 | x | x | x | x | - | Barcode |
| ISO008-17 | ITV258 | IS | Parauapebas | N3 | x | x | x | x | - | Barcode |
| ISO009-17 | ITV259 | IS | Parauapebas | N3 | x | x | - | x | - | Barcode |
| ISO010-17 | ITV260 | IS | Parauapebas | N3 | x | x | - | x | - | Barcode |
| ISO011-17 | ITV274 | IS | Parauapebas | N4WS | x | - | - | x | - | Barcode |
| ISO012-17 | ITV275 | IS | Parauapebas | N4WS | x | - | - | x | - | Barcode |
| ISO013-17 | ITV276 | IS | Parauapebas | N4WS | - | x | - | x | x | Barcode |
| ISO014-17 | ITV277 | IS | Parauapebas | N4WS | x | - | x | x | - | Barcode |
| ISO015-17 | ITV289 | IS | Parauapebas | N6 | x | x | x | x | x | Barcode |
| ISO016-17 | ITV292 | IS | Parauapebas | N6 | x | x | - | - | - | Barcode |
| ISO017-17 | ITV293 | IS | Parauapebas | N6 | x | - | - | x | - | Barcode |
| ISO018-17 | ITV294 | IS | Parauapebas | N7 | x | - | x | - | - | Barcode |
| ISO019-17 | ITV306 | IS | Canaa | Tarzan | x | x | x | x | - | Barcode |
| ISO020-17 | ITV308 | IS | Canaa | Tarzan | x | - | - | - | - | Barcode |
| ISO021-17 | ITV325 | IS | Canaa | Bocaina | x | x | x | x | - | Barcode |
| ISO022-17 | ITV333 | IS | Canaa | S11B | x | - | - | x | - | Barcode |
| ISO023-17 | ITV335 | IS | Canaa | S11B | x | x | x | x | - | Barcode |
| ISO024-17 | ITV349 | IC | Canaa | S11D | x | x | x | x | - | Barcode |
| ISO025-17 | ITV350 | IC | Canaa | S11D | x | x | x | x | - | Barcode |
| ISO026-17 | ITV351 | IC | Canaa | S11D | x | x | x | x | - | Barcode |
| ISO027-17 | ITV404 | IS | Canaa | Bocaina | x | x | - | - | - | Barcode |
| ISO028-17 | ITV405 | IS | Canaa | Bocaina | x | x | - | - | - | Barcode |
| ISO029-17 | ITV406 | IS | Canaa | Bocaina | x | x | - | - | - | Barcode |
| ISO030-17 | ITV407 | IS | Canaa | Bocaina | x | x | - | - | - | Barcode; SNPs |
| ISO031-17 | ITV408 | IS | Canaa | Bocaina | x | - | - | - | - | Barcode |
| ISO032-17 | ITV409 | IS | Canaa | Bocaina | x | x | - | - | - | Barcode |
| ISO033-17 | ITV410 | IS | Canaa | Bocaina | x | x | - | - | - | Barcode |
| ISO034-17 | ITV411 | IS | Canaa | Bocaina | x | x | - | - | - | Barcode; chloroplast |
| ISO035-17 | ITV412 | IS | Canaa | Bocaina | x | x | - | - | - | Barcode |
| ISO036-17 | ITV413 | IS | Canaa | Bocaina | x | x | - | - | - | Barcode |
| ISO037-17 | ITV414 | IS | Canaa | Bocaina | x | x | - | - | - | Barcode; SNPs |
| ISO038-17 | ITV415 | IS | Canaa | Bocaina | x | x | - | - | - | Barcode |
| ISO039-17 | ITV416 | IS | Canaa | Bocaina | x | x | - | - | - | Barcode |
| ISO040-17 | ITV417 | IS | Canaa | Bocaina | x | x | - | - | - | Barcode |
| ISO041-17 | ITV418 | IS | Canaa | Bocaina | - | x | - | - | - | Barcode |
| ISO042-17 | ITV419 | IS | Canaa | Bocaina | x | x | - | - | - | Barcode |
| ISO043-17 | ITV420 | IS | Canaa | Bocaina | x | x | - | - | - | Barcode |
| ISO044-17 | ITV421 | IS | Canaa | Bocaina | x | x | - | - | - | Barcode |
| ISO045-17 | ITV422 | IS | Canaa | Bocaina | x | x | - | - | - | Barcode |
| ISO046-17 | ITV423 | IS | Canaa | Bocaina | x | x | - | - | - | Barcode |
| ISO047-17 | ITV1757 | IS | Canaa | Tarzan | x | - | - | - | - | Barcode |
| ISO049-17 | ITV1997 | IC | Canaa | S11D | x | - | - | - | - | Barcode; SNPs |
| ISO050-17 | ITV1998 | IC | Canaa | S11D | x | - | - | - | - | Barcode |
| ISO051-17 | ITV1999 | IC | Canaa | S11D | x | - | - | - | - | Barcode |
| ISO052-17 | ITV2000 | IC | Canaa | S11D | x | - | - | - | - | Barcode |
| ISO053-17 | ITV2001 | IC | Canaa | S11D | x | - | - | - | - | Barcode |
| ISO054-17 | ITV2002 | IC | Canaa | S11D | x | - | - | - | - | Barcode |
| ISO055-17 | ITV2003 | IC | Canaa | S11D | x | - | - | - | - | Barcode |
| ISO056-17 | ITV2004 | IC | Canaa | S11D | x | - | - | - | - | Barcode |
| ISO057-17 | ITV2005 | IC | Canaa | S11D | x | - | - | - | - | Barcode |
| ISO058-17 | ITV2006 | IC | Canaa | S11D | x | - | - | - | - | Barcode |
| ISO059-17 | ITV2007 | IC | Canaa | S11D | x | - | - | - | - | Barcode |
| ISO060-17 | ITV2008 | IC | Canaa | S11D | x | - | - | - | - | Barcode; chloroplast |
| ISO061-17 | ITV2009 | IC | Canaa | S11D | x | - | - | - | - | Barcode; SNPs |
| ISO062-17 | ITV2010 | IC | Canaa | S11D | x | - | - | - | - | Barcode |
| ISO063-17 | ITV2011 | IC | Canaa | S11D | x | - | - | - | - | Barcode |
| ISO064-17 | ITV2012 | IC | Canaa | S11D | x | - | - | - | - | Barcode; SNPs |
| ISO067-17 | ITV2015 | IC | Canaa | S11D | x | - | - | - | - | Barcode |
| ISO070-17 | ITV2097 | IC | Canaa | S11D | x | - | - | - | - | Barcode |
| ISO071-17 | ITV2098 | IC | Canaa | S11D | x | - | - | - | - | Barcode |
| ISO072-17 | ITV2101 | IC | Canaa | S11D | x | - | - | - | - | Barcode |
| ISO074-17 | ITV2103 | IC | Canaa | S11D | x | - | - | - | - | Barcode |
| ISO075-17 | ITV2104 | IC | Canaa | S11D | x | - | - | - | - | Barcode |
| ISO076-17 | ITV2106 | IC | Canaa | S11D | x | - | - | - | - | Barcode |
| ISO077-17 | ITV2107 | IC | Canaa | S11D | x | - | - | - | - | Barcode; SNPs |
| ISO078-17 | ITV2108 | IC | Canaa | S11D | x | - | - | - | - | Barcode |
| ISO079-17 | ITV2109 | IC | Canaa | S11D | x | - | - | - | - | Barcode |
| ISO081-17 | ITV2180 | IS | Parauapebas | N4SW | x | - | - | - | - | Barcode |
| ISO084-17 | ITV2773 | IS | Canaa | Bocaina | x | x | - | - | - | Barcode |
| ISO085-17 | ITV2774 | IS | Canaa | Bocaina | x | - | - | - | - | Barcode |
| ISO086-17 | ITV2775 | IS | Canaa | Bocaina | x | x | - | - | - | Barcode |
| ISO087-17 | ITV2776 | IS | Canaa | Bocaina | x | x | - | - | - | Barcode |
| ISO088-17 | ITV2777 | IS | Canaa | Bocaina | x | x | - | - | - | Barcode |
| ISO089-17 | ITV2778 | IS | Parauapebas | N4WS | x | x | - | - | - | Barcode |
| ISO090-17 | ITV2779 | IS | Parauapebas | N4WS | x | x | - | - | - | Barcode |
| ISO091-17 | ITV2780 | IS | Parauapebas | N4WS | x | x | - | - | - | Barcode |
| ISO092-17 | ITV2781 | IS | Parauapebas | N4WS | x | x | - | - | - | Barcode; SNPs |
| ISO093-17 | ITV2782 | IS | Parauapebas | N4WS | x | x | - | - | - | Barcode |
| ISO094-17 | ITV2783 | IS | Parauapebas | N4WS | x | x | - | - | - | Barcode |
| ISO095-17 | ITV2784 | IS | Parauapebas | N4WS | x | x | - | - | - | Barcode |
| ISO096-17 | ITV2785 | IS | Parauapebas | N4WS | x | x | - | - | - | Barcode |
| ISO097-17 | ITV2786 | IS | Parauapebas | N4WS | x | x | - | - | - | Barcode |
| ISO098-17 | ITV2787 | IS | Parauapebas | N6 | x | x | - | - | - | Barcode |
| ISO099-17 | ITV2788 | IS | Parauapebas | N6 | x | - | - | - | - | Barcode; SNPs |
| ISO100-17 | ITV2789 | IS | Parauapebas | N6 | x | x | - | - | - | Barcode |
| ISO101-17 | ITV2790 | IS | Parauapebas | N6 | x | x | - | - | - | Barcode |
| ISO102-17 | ITV2791 | IS | Parauapebas | N6 | x | - | - | - | - | Barcode |
| ISO103-17 | ITV2792 | IS | Parauapebas | N6 | x | x | - | - | - | Barcode |
| ISO104-17 | ITV2793 | IS | Parauapebas | N6 | x | x | - | - | - | Barcode |
| ISO105-17 | ITV2794 | IS | Parauapebas | N6 | x | x | - | - | - | Barcode |
| ISO106-17 | ITV2795 | IS | Parauapebas | N6 | x | x | - | - | - | Barcode |
| ISO107-17 | ITV2796 | IS | Parauapebas | N6 | x | - | - | - | - | Barcode |
| ISO108-17 | ITV2797 | IS | Parauapebas | N6 | x | x | - | - | - | Barcode |
| ISO109-17 | ITV2798 | IS | Parauapebas | N6 | x | x | - | - | - | Barcode |
| ISO110-17 | ITV2799 | IS | Parauapebas | N6 | x | x | - | - | - | Barcode |
| ISO111-17 | ITV2800 | IS | Parauapebas | N6 | x | - | - | - | - | Barcode |
| ISO112-17 | ITV2801 | IS | Parauapebas | N6 | x | x | - | - | - | Barcode |
| ISO113-17 | ITV2802 | IS | Parauapebas | N6 | x | x | - | - | - | Barcode |
| ISO114-17 | ITV2803 | IS | Parauapebas | N6 | x | x | - | - | - | Barcode |
| ISO115-17 | ITV2804 | IS | Parauapebas | N7 | x | x | - | - | - | Barcode |
| ISO116-17 | ITV2805 | IS | Parauapebas | N7 | x | x | - | - | - | Barcode |
| ISO117-17 | ITV2806 | IS | Parauapebas | N7 | x | x | - | - | - | Barcode |
| ISO118-17 | ITV2807 | IS | Parauapebas | N7 | x | - | - | - | - | Barcode |
| ISO119-17 | ITV2808 | IS | Parauapebas | N7 | x | x | - | - | - | Barcode |
| ISO120-17 | ITV2809 | IC | Canaa | S11D | x | x | - | - | - | Barcode |
| ISO121-17 | ITV2810 | IC | Canaa | S11D | x | x | - | - | - | Barcode |
| ISO122-17 | ITV2811 | IC | Canaa | S11D | x | x | - | - | - | Barcode |
| ISO123-17 | ITV2812 | IC | Canaa | S11D | x | x | - | - | - | Barcode |
| ISO124-17 | ITV2813 | IC | Canaa | S11D | x | x | - | - | - | Barcode |
| ISO125-17 | ITV2814 | IC | Canaa | S11D | x | x | - | - | - | Barcode |
| ISO126-17 | ITV2815 | IC | Canaa | S11D | x | x | - | - | - | Barcode |
| ISO127-17 | ITV2816 | IS | Canaa | S11B | x | x | - | - | - | Barcode |
| ISO128-17 | ITV2817 | IS | Canaa | S11B | x | x | - | - | - | Barcode |
| ISO129-17 | ITV2818 | IS | Canaa | S11B | x | x | - | - | - | Barcode |
| ISO130-17 | ITV2819 | IS | Canaa | S11B | x | x | - | - | - | Barcode |
| ISO131-17 | ITV2820 | IS | Canaa | S11B | x | - | - | - | - | Barcode |
| ISO132-17 | ITV2821 | IS | Canaa | S11B | x | x | - | - | - | Barcode |
| ISO133-17 | ITV2822 | IS | Canaa | S11B | x | x | - | - | - | Barcode |
| ISO134-17 | ITV2823 | IS | Canaa | Tarzan | x | x | - | - | - | Barcode |
| ISO135-17 | ITV2824 | IS | Canaa | Tarzan | x | x | - | - | - | Barcode |
| ISO136-17 | ITV2825 | IS | Canaa | Tarzan | x | x | - | - | - | Barcode |
| ISO137-17 | ITV2826 | IS | Canaa | Tarzan | x | x | - | - | - | Barcode |
| ISO138-17 | ITV2827 | IS | Canaa | Tarzan | x | x | - | - | - | Barcode |
| ISO139-17 | ITV2828 | IS | Canaa | Tarzan | x | x | - | - | - | Barcode |
| ISO140-17 | ITV2829 | IS | Canaa | Tarzan | x | x | - | - | - | Barcode |
| ISO141-17 | ITV2830 | IS | Canaa | Cristalino | x | x | - | - | - | Barcode |
| ISO142-17 | ITV2831 | IS | Canaa | Cristalino | x | x | - | - | - | Barcode |
| ISO143-17 | ITV2832 | IS | Canaa | Cristalino | x | x | - | - | - | Barcode |
| ISO144-17 | ITV2833 | IS | Canaa | Cristalino | x | x | - | - | - | Barcode |
| ISO145-17 | ITV2834 | IS | Canaa | Cristalino | x | x | - | - | - | Barcode |
| ISO146-17 | ITV2835 | IS | Canaa | Cristalino | x | x | - | - | - | Barcode |
| ISO147-17 | ITV2836 | IS | Canaa | Cristalino | x | - | - | - | - | Barcode |
| ISO148-17 | ITV2837 | IS | Canaa | Cristalino | x | x | - | - | - | Barcode |
| ISO149-17 | ITV2838 | IS | Canaa | Cristalino | x | x | - | - | - | Barcode |
| ISO150-17 | ITV2839 | IS | Canaa | Cristalino | x | x | - | - | - | Barcode |
| ISO151-17 | ITV3819 | IC | Canaa | S11D | x | - | - | - | - | Barcode; Morphometry |
| ISO152-17 | ITV3820 | IC | Canaa | S11D | x | - | - | - | - | Barcode; Morphometry |
| ISO153-17 | ITV3821 | IC | Canaa | S11D | x | - | - | - | - | Barcode; Morphometry |
| ISO154-17 | ITV3822 | IC | Canaa | S11D | x | - | - | - | - | Barcode; Morphometry |
| ISO155-17 | ITV3823 | IC | Canaa | S11D | x | - | - | - | - | Barcode; Morphometry |
| ISO156-17 | ITV3824 | IC | Canaa | S11D | x | - | - | - | - | Barcode; Morphometry |
| ISO157-17 | ITV3825 | IC | Canaa | S11D | x | - | - | - | - | Barcode; Morphometry |
| ISO158-17 | ITV3826 | IC | Canaa | S11D | x | - | - | - | - | Barcode; Morphometry |
| ISO159-17 | ITV3827 | IC | Canaa | S11D | x | - | - | - | - | Barcode; Morphometry |
| ISO160-17 | ITV3828 | IC | Canaa | S11D | x | - | - | - | - | Barcode; Morphometry |
| ISO161-17 | ITV4560 | IS | Canaa | S11B | x | - | - | - | - | Barcode; Morphometry |
| ISO162-17 | ITV4561 | IS | Canaa | S11B | x | - | - | - | - | Barcode; Morphometry |
| ISO163-17 | ITV4562 | IS | Canaa | S11B | x | - | - | - | - | Barcode; Morphometry |
| ISO164-17 | ITV4563 | IS | Canaa | S11B | x | - | - | - | - | Barcode; Morphometry |
| ISO165-17 | ITV4564 | IS | Canaa | S11B | x | - | - | - | - | Barcode; Morphometry |
| ISO166-17 | ITV4565 | IS | Canaa | S11B | x | - | - | - | - | Barcode; Morphometry |
| ISO167-17 | ITV4566 | IS | Canaa | S11B | x | - | - | - | - | Barcode; Morphometry |
| ISO168-17 | ITV4567 | IS | Canaa | S11B | x | - | - | - | - | Barcode; Morphometry |
| ISO169-17 | ITV4568 | IS | Canaa | S11D | x | - | - | - | - | Barcode; Morphometry |
| ISO170-17 | ITV4569 | IS | Canaa | S11D | x | - | - | - | - | Barcode; Morphometry |
| ISO171-17 | ITV4570 | IS | Canaa | S11D | x | - | - | - | - | Barcode; Morphometry |
| ISO172-17 | ITV4571 | IS | Canaa | S11D | x | - | - | - | - | Barcode; Morphometry |
| ISO173-17 | ITV4572 | IS | Canaa | S11D | x | - | - | - | - | Barcode; Morphometry |
| ISO174-17 | ITV4573 | IS | Canaa | S11D | x | - | - | - | - | Barcode; Morphometry |
| NA | ITV3808 | IC | Canaa | S11D | - | - | - | - | - | Chloroplast |

Specimen data can be access by Process ID on Bold database. All records with Process ID were deposited in the Genbank. Canaa – Canaã dos Carajás, Tarzan - Serra do Tarzan, Bocaina - Serra da Bocaina. Region indicates the location of sampling (see Figure 1). The species are coded as: *Isoetes cangae* – IC, *Isoetes serracarajensis* – IS. Barcode, Morphometry, Chloroplast and SNPs indicate if the specimen was used for DNA barcode production, morphometric analysis, chloroplast genome sequencing and SNP analysis, respectively.
