# Supplementary material for: Quillworts from the Amazon: A multidisciplinary populational study on Isoetes serracarajensis and Isoetes cangae
Source: PLoS One. 2018 Aug 8;13(8):e0201417. doi: 10.1371/journal.pone.0201417 (PMC6082551; doi:10.1371/journal.pone.0201417)
Supplement: S1 File — (DOCX) [file pone.0201417.s007.docx]

Supplemental File S3: Software and command lines for bioinformatics analysis.

1. **FastxToolKit** : Trimming and quality filters

Quality treatment trimmed reads with quality lower than 20 and shorter than 100bp. Filtering removed reads with less than 80% content with quality at least 20.

>fastq_quality_trimmer -Q 33 -t 20 -l 100 -i Isoetes.ITV411.fastq -o Isoetes.ITV144.trim.fastq

>fastq_quality_filter -Q 33 -q 20 -p 80 -i Isoetes.ITV144.trim.fastq -o Isoetes.ITV144.trim.filter.fastq

2. **Prinseq:** Input and filter stats (Input sequences, Input bases, Input mean length, good and bad sequences)

>prinseq-lite.pl -verbose -fastq Isoetes.ITV411.trim.filter.fastq -ns_max_n 0 -out_good test_no_ns -out_bad test_with_ns

3. **Arc**: Mapping using a reference genome

Run in ARC terminal inside the folder containing the ARC_config.txt with the following information:

## Configuration options start with a single "#" and use

## Name=value pairs

##

## Data Columns define samples:

## Sample_ID: ITV411

## FileName: path for fasta/fastq file

## FileType: PE1, PE2, or SE

## FileFormat: fasta or fastq

# reference= IsoetesFlaccidaNC014675.fa

# numcycles=10

# mapper=bowtie2

# assembler=spades

# only-assembler=False

# nprocs=5

# format=fastq

# verbose=True

# urt=True

# map_against_reads=False

# assemblytimeout=30

# bowtie2_k=5

# rip=True

# subsample=1

# maskrepeats=True

# sloppymapping=True

# sra=False

Sample_ID FileName FileType

Sample1 Isoetes.ITV411.trim.filter.good.fastq SE

4. **Spades**: Assembly

Reads used in assembly were mapped against of I. flaccida chloroplast using Geneious R10

spades.py --careful --iontorrent --only-assembler -k 31,39,47,55 -t 5 -s Isoetes.ITV411.trim.filter.good.map.fastq -o assembliesSPADE

5. **Mauve** - Ordering contigs

java -Xmx500m -cp Mauve.jar org.gel.mauve.contigs.ContigOrderer -output results_dir -ref '/home/manager/Documents/Isoetes_genoma/ IsoetesFlaccidaNC014675.fa -draft '/home/manager/Documents/Isoetes_genoma/Geneious/SPADEs_Geneious/ITV411.contigs.fasta'

6. **Quast** – Comparative analysis (statistics)

./quast.py 'ITV411.contigs.fasta IsoetesFlaccidaNC014675.fa '

1. **Bowtie2** - Reads mapping of I. cangae against *Isoetes serracarajensis* chloroplast (ITV411) for SNPs analysis

> bowtie2-build ITV411_chloroplast.fasta ITV411_index

> bowtie2 -p 8 -x ITV411_index -1 2008_S5_R1_001.fastq -2 2008_S5_R2_001.fastq -S 2008_aligned_to_411.sam --al-conc paired_aligned_to_411.fastq --un-conc paired_unaligned_to_411.fastq

1. **Samtools 1.3.1** - Generating SAM and BAM files

>samtools view --threads 24 -s -b 2008_aligned_to_411.sam -o 2008_aligned_to_411.bam

>samtools sort --threads 24 -o 2008_aligned_to_411_ordenado.bam

2008_aligned_to_411.bam

>samtools mpileup -gu -Q 10 -t DP,DPR -f ITV411_chloroplast.fasta -b inputBamFiles.txt BCF_test_sp1.bcf

1. 9. **BCFtools** - Generating VCF file

>bcftools call -cv BCF_test_sp1.bcf -> VCF_test_sp2.vcf
