# Supplementary figures and images for: Quillworts from the Amazon: A multidisciplinary populational study on Isoetes serracarajensis and Isoetes cangae
Source: PLoS One. 2018 Aug 8;13(8):e0201417. doi: 10.1371/journal.pone.0201417 (PMC6082551; doi:10.1371/journal.pone.0201417)

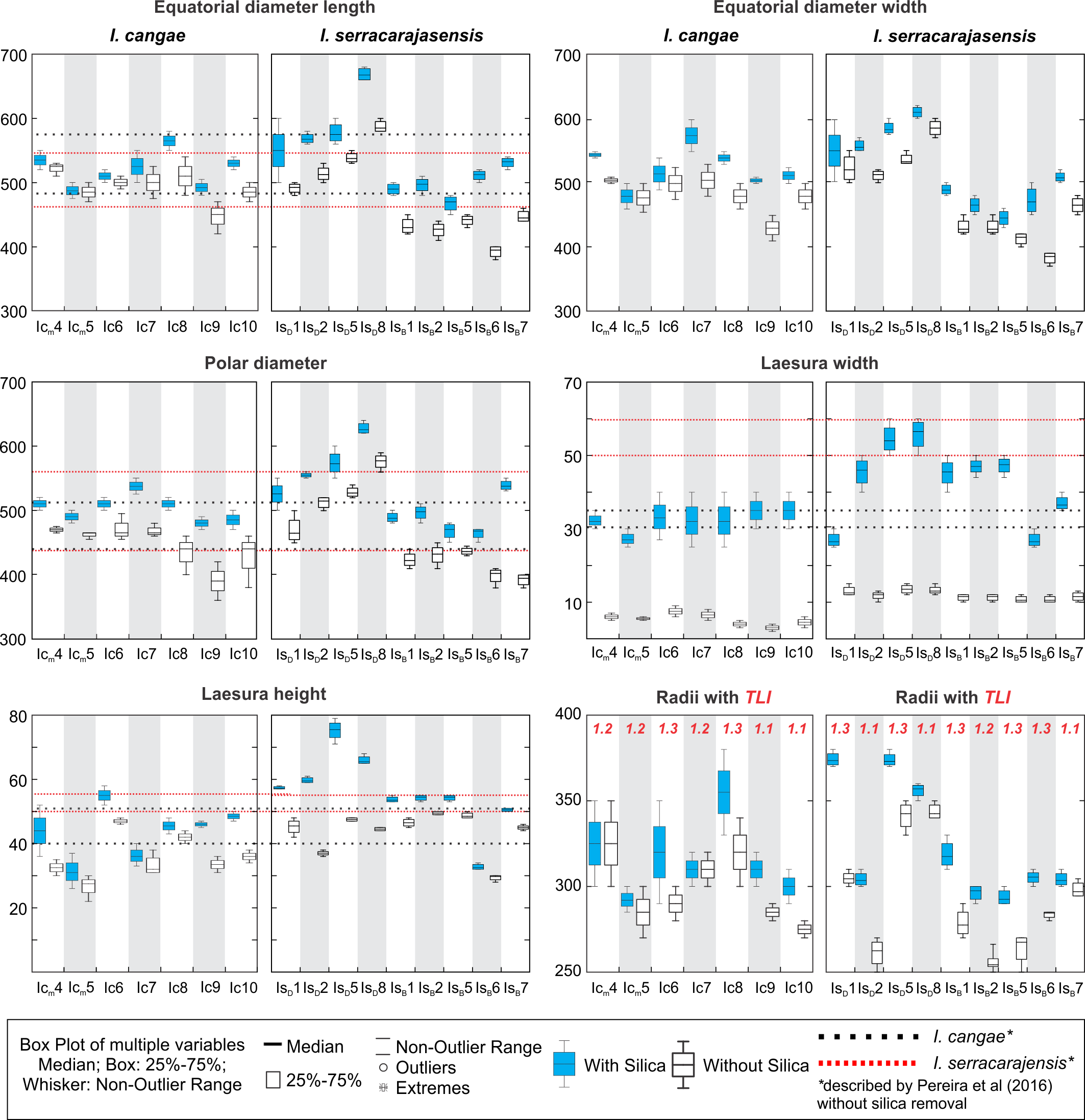

Supplement: S1 Fig — Box plots of equatorial diameter length, equatorial diameter width, polar diameter, leasure width, leasura hight, and radii with TLI for I. cangae and I. serracarajensis with and without silica. All units in the y-axis are in μm. (TIF) [file pone.0201417.s008.tif]

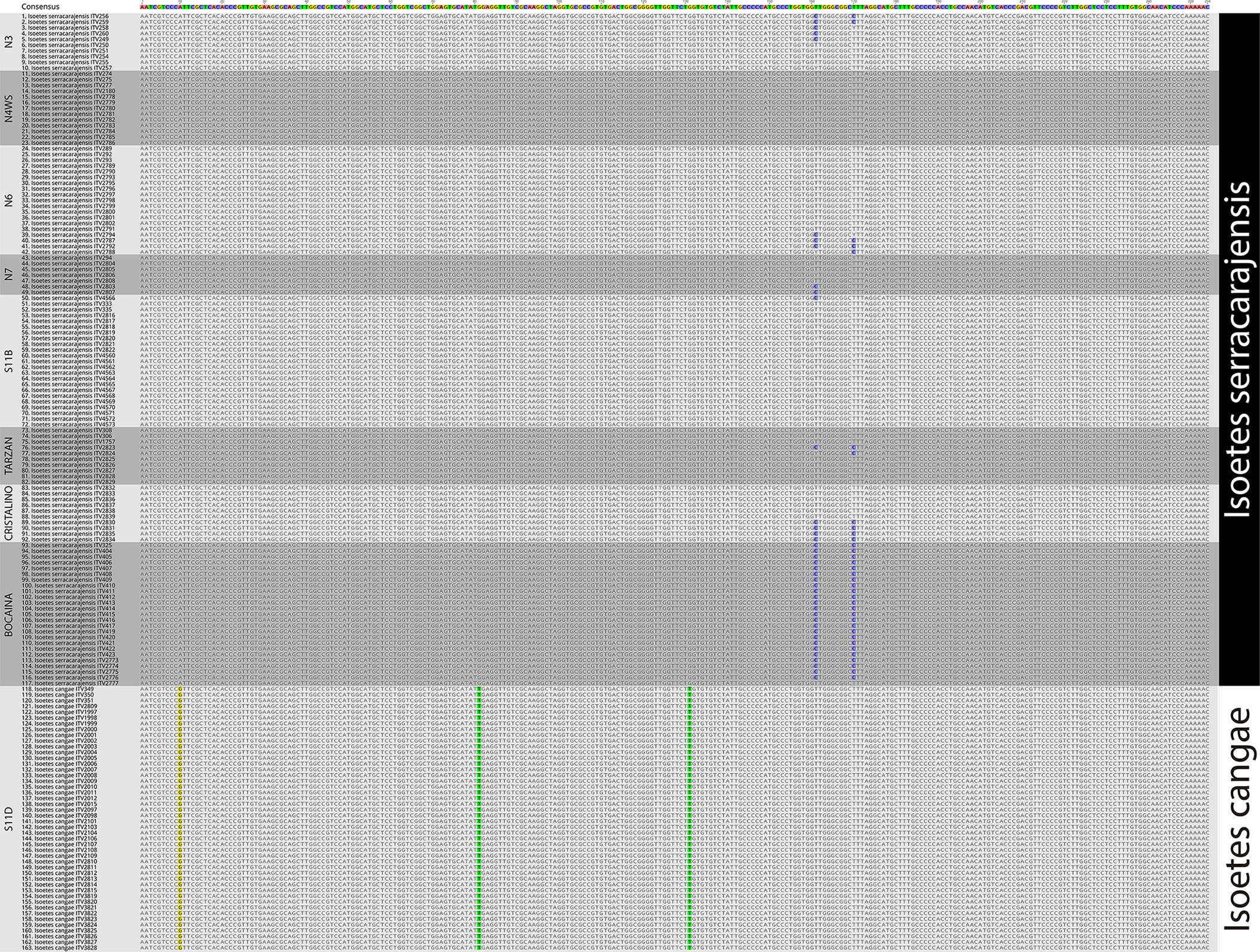

Supplement: S2 Fig — The species and the sampled locations are shown in the left. Polymorphic sites are marked in green and yellow for I. cangae and in blue for I. serracarajensis. (TIF) [file pone.0201417.s009.tif]
